# A Novel Peptide, HS1002, Enhances Antitumor Activity via Dual Targeting of the GnRH Receptor and Human Telomerase Reverse Transcriptase in Prostate Cancer Cells

**DOI:** 10.1002/mco2.70630

**Published:** 2026-02-15

**Authors:** Jae Hyeon Park, Joo Chan Lee, Swati Sharma, Chunxue Jiang, Haeun Lee, Hyun‐Ju Park, Hyung Sik Kim

**Affiliations:** ^1^ School of Pharmacy Sungkyunkwan University Suwon Republic of Korea

**Keywords:** GnRHR, HS1002, hTERT, immune response, prostate cancer

## Abstract

Human telomerase reverse transcriptase (hTERT) is overexpressed in most human cancers and is an important target for cancer therapy. In this study, HS1002 was synthesized based on the amino acid sequences of gonadotropin‐releasing hormone (GnRH) and hTERT. This study aimed to evaluate HS1002's anticancer activity and its effects on the gonadotropin‐releasing hormone receptor (GnRHR) and hTERT in prostate cancer cells. HS1002 increased cytosolic calcium influx in GnRHR‐overexpressing HEK293 cells and showed specific molecular docking interactions with GnRHR. Compared with prostate cancer cell lines, HS1002 exhibited the highest cytotoxicity against LNCaP cells. The hTERT expression correlated with telomerase activity was suppressed by HS1002, resulting in reduced metastasis and increased apoptosis and autophagy. Additionally, HS1002 suppressed c‐Myc and ERK protein expressions in LNCaP cells. Furthermore, HS1002 inhibited tumor growth and downregulated hTERT expression in the xenograft model tumor tissues. HS1002/IL‐2‐pretreated PBMCs also exhibited potent cytotoxicity toward LNCaP cells. In addition, HS1002 increased the production of granzyme B and IFN‐γ in CD8^+^ T cells in MC38 syngeneic mice. These findings demonstrate that HS1002 suppresses prostate cancer cell growth and induces anticancer immunity, suggesting its potential as a novel therapeutic agent against prostate cancer.

## Graphical abstract

HS1002, a new peptide targeting GnRHR and hTERT, demonstrates significant Anticancer activity in prostate cancer cells. The mechanism of HS1002 can be attributed to the reduction of serum testosterone levels by desensitization of GnRHR, the downregulation of hTERT expression and telomerase activity, and the enhancement of Anticancer immunity. These findings suggest HS1002's potential as a novel therapeutic agent against prostate cancer.

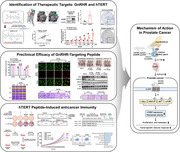

## 1 Introduction

Telomeres have a crucial function in the protection of chromosomal ends by preventing DNA damage. During eukaryotic cell division, telomerase acts to preserve telomere length by adding the repetitive sequence 5′‐TTAGGG‐3′ [1]. Activation of telomerase is detected in almost 90% of human malignancies, including prostate and breast cancers, and plays a crucial role in enabling limitless cell proliferation [2]. Enhanced telomerase activity in cancers not only suppresses telomere shortening, but also induces apoptotic resistance, stemness, and metastatic potential, which are hallmarks of cancer [3]. The tightly regulated expression of the catalytic subunit of telomerase, known as human telomerase reverse transcriptase (hTERT), determines telomerase activity. Although the *hTERT* gene remains silent in somatic cells after birth, germline, stem, and malignant cells exhibit elevated levels of hTERT expression [4]. In this regard, hTERT represents an optimal therapeutic target for human cancers. Targeting hTERT using inhibitors or immunotherapy is a promising therapeutic strategy [5].

While various treatments, such as chemotherapy, radiation, and prostatectomy, are available, hormonal therapy remains an essential treatment option for patients with symptomatic metastatic prostate cancer. Various gonadotropin‐releasing hormone (GnRH) analogs have been used as first‐line hormonal therapies to reduce testosterone levels [6]. GnRH receptor (GnRHR) activation by GnRHR agonists induces a multifaceted signaling network that involves the calcium and cyclic AMP (cAMP) pathways in pituitary gonadotrope cells [7]. GnRH also modulates cell growth and metastasis in malignancies. GnRHR is expressed in prostate cancer tissues and interacts with GnRH analogs to induce antiproliferative, proapoptotic, and antimetastatic effects [8]. The expression of GnRHR in multiple tumor types highlights its potential as a target for GnRH analog therapy.

GV1001, a 16‐amino acid (residues 611–626) derived from the hTERT protein, has been developed as a cancer vaccine targeting several solid tumors. The hTERT:611–626 peptide vaccination generates CD4 clones that recognize naturally processed hTERT, demonstrating therapeutic potential and safety across clinical trials [9]. Its adaptability to bind to molecules encoded by multiple alleles of all three HLA class II loci without patient HLA typing enables efficient hTERT‐specific T cell responses, encompassing both cytotoxic CD8^+^ T lymphocyte (CTL) and T helper responses [10]. A recent study identified that the hTERT:611–626 peptide is structurally similar to three GnRH analogues (GnRH, leuprolide, and cetrorelix) in its 9‐amino acid sequence (Ile–Phe–Arg–Leu–Arg–Ser–Thr–Leu–Leu), which includes the Leu–Arg region for ligand selectivity [11].

This study aimed to demonstrate the hTERT suppression‐mediated anticancer effects of HS1002 in prostate cancer, both in vitro and in vivo models. It was also determined that HS1002 could function as a GnRHR agonist and induce GnRHR downstream signaling. Moreover, HS1002 promoted antitumor immunity by activating CD8^+^ T cells. Collectively, these results highlight that HS1002 is a promising candidate for prostate cancer therapy.

## 2 Results

### 2.1 HS1002 Targets GnRHR to Induce GnRH Downstream Signaling Pathway

**FIGURE 1:**
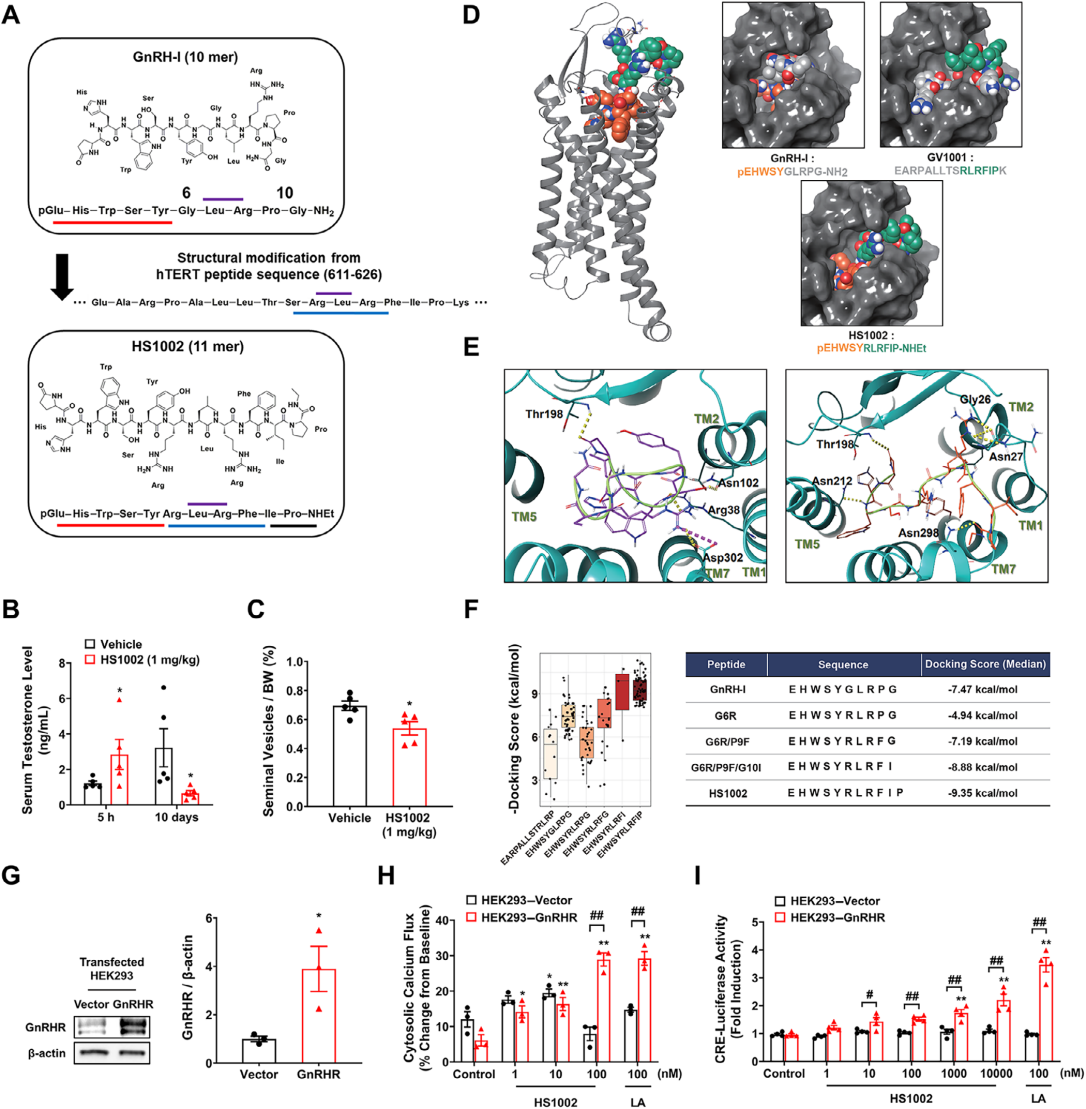
Peptide docking analysis and evaluation of GnRHR activation by HS1002. (A) Peptide design for targeting GnRHR and hTERT in prostate cancer. Each colored residue corresponds to the GnRH or hTERT peptide. (B) The serum testosterone levels of BALB/c mice administered with either a vehicle or HS1002 (1 mg/kg) were measured. (C) Percentage of seminal vesicles/body weight. The seminal vesicles and testes were obtained from BALB/c mice following 10 days of administration of the vehicle or HS1002. (D) A side view of the binding pose of HS1002 and top views of the binding pose of each peptide. The molecular surface of GnRHR is colored gray, and the peptides are represented as CPK (space‐filling) models. The identical sequences between peptides are differentiated by colors. (E) The intermolecular interactions of GnRH (left, magenta carbon) and HS1002 (right, orange carbon) within the binding region of the receptor. The oxygen and nitrogen atoms are depicted as red and blue, respectively. The backbone of the peptide ligand is displayed in a green tube. Key amino acid residues within the binding site are labeled and rendered in line form (carbon atoms in cyan). Intermolecular interactions with the ligand are presented by dashed lines: hydrogen boning is yellow, and the salt bridge is magenta. (F) Comparison of docking scores of various mutants of GnRH‐I and HS1002, each point representing a docking score obtained from flexible docking. The median value of the docking score of each peptide is listed in the table. (G) GnRHR protein expression in HEK293–Vector or GnRHR cells. β‐Actin was used as a loading control. Effect of HS1002 or LA on (H) cytosolic calcium flux change and (I) CRE‐luciferase activity in HEK293–Vector or GnRHR cells. LA was used as a positive control. The values represent the means ± SD. *p < 0.05 and **p < 0.01 versus control group; ^#^ *p* < 0.05 and ^##^ *p* < 0.01 between two groups. LA, leuprolide acetate.

A new peptide, HS1002, was designed by introducing amino acid substitutions and cleavage around the Leu–Arg region of the hTERT:611–626 peptide to target GnRHR. HS1002 is a hybrid undecapeptide consisting of the N‐terminal five amino acid residues (E^1^H^2^W^3^S^4^Y^5^) from GnRH and the following six amino acid sequences (R^6^L^7^R^8^F^9^I^10^P^11^) from GV1001 (Figure 1A). Specifically, HS1002 was constructed by connecting the Arg–Leu–Arg–Phe–Ile sequence from the hTERT:611–626 peptide to the pGlu–His–Trp–Ser–Tyr sequence involved in GnRHR activation from the GnRH peptide after cleavage of the Gly [6] region susceptible to peptide degradation [12]. GnRHR agonists activate the release of luteinizing hormone (LH) and follicle‐stimulating hormone by binding to GnRHR receptors found on the pituitary gonadotropes, which in turn stimulates the synthesis of steroid hormones from gonads [13]. However, continuous exposure to GnRHR agonists leads to the suppression of GnRHR sensitivity and eventually reduces testosterone synthesis [14]. Based on the HS1002 peptide design strategy, the serum testosterone concentration in male mice was tested after repeated subcutaneous injections of HS1002 to determine whether HS1002 acts as a GnRHR ligand (Figure S1A). Serum testosterone levels showed a 2.64‐fold increase 5 h after the first HS1002 injection, compared with those in the vehicle‐injected group. However, the serum testosterone levels and seminal vesicle weights were notably reduced at 10 days following repeated administration of HS1002 compared with those in the vehicle‐injected group (Figures 1B,C and S1B).

Docking analysis was performed to demonstrate the molecular interaction of HS1002 with GnRHR. The ligand‐binding site for GnRHR was assigned for docking, based on published literature [11, 15, 16]. In Figure 1D, the selected docking poses for GV1001, GnRH, and HS1002 are displayed. All peptides are well‐fitted into the known ligand‐binding site of GnRHR. The natural ligand GnRH formed a beta‐turn conformation and occupied the inside of the binding site. The N‐terminal pEHWSY motif of HS1002 occupied the inside of the binding pocket, while the following RLRFIP–NHEt extended to the extracellular side of GnRHR. HS1002 formed a nonbulged linear conformation (Figure 1E, right) and occupied the entire Y‐shaped binding pocket. Similarly, the RLRFIPK motif of GV1001 reached out to the extracellular side of the receptor. To examine the docked pose of GnRH in detail (Figure 1E, left), the N‐terminal lactone carbonyl oxygen of pGlu1 (pyroglutamic acid) interacts with the sidechain OH of Thr198 and the backbone NH of Trp3 with Arg38^1.35^ inside the active site of GnRHR by forming H‐bonds. Additionally, the sidechain OH of Ser4 interacts with the sidechain carbonyl of Asn102^2.65^. The sidechain guanidium of Arg8 forms a salt bridge with Asp302^7.32^, which is one of the critical interactions for maintaining the active conformation of GnRHR. These interactions may help GnRH fit into the active site in a beta‐turn conformation. It was reported that the N‐terminus of GnRH is an active center, and the modification of the first three amino acid residues of the natural peptide can give antagonistic activity. Thus, the rest plays a critical role only in the binding process. The docking pose of HS1002 (Figure 1E, right) revealed that the pGlu1 residue anchored at the same binding site as GnRH, forming an H‐bond with Thr198. In addition, the backbone NH of His2 interacts with the side chain of Asn212^5.39^. The interactions of HS1002 differentiated from those of GnRH were identified; the sidechain guanidium group of Arg8 forms a H‐bond network with the backbone carbonyl oxygen atoms of Gly26 and Asn27, and the C‐terminal amide carbonyl of Pro11–NHEt forms a H‐bond with the side chain NH of Asn298. In relation to the binding pose of HS1002, we investigated the effect of the R^6^L^7^R^8^F^9^I^10^ motif adopted from GV1001 on the binding affinity by estimating the relative binding energy scores of several mutant peptides. As listed in Figure 1F, the amino acid sequence of GnRH was consecutively mutated from the Gly6 to the terminal Gly10, except for Lys7 and Arg8. The binding energy was gradually decreased by replacing G^6^L^7^R^8^P^9^G^10^ (−7.47 kcal/mol) with R^6^L^7^R^8^F^9^I^10^ and R^6^L^7^R^8^F^9^I^10^P^11^ (−8.88 and −9.35 kcal/mol, respectively), implicating that binding affinity would be significantly increased in HS1002 containing the R^6^L^7^R^8^F^9^I^10^P^11^ motif in comparison with GnRH. These results from comparative docking analyses of HS1002 and various homologous peptides strongly support the biological activity of HS1002 as a GnRHR agonist.

To confirm whether HS1002 induced the downstream signaling pathway of GnRHR, a GnRHR‐overexpressing HEK293 cell line was constructed (Figure 1G). The effects of HS1002 and LA on Gαq‐stimulated intracellular calcium release and Gαs‐mediated cAMP activity were investigated in HEK293–GnRHR cells. The calcium flux signal, measured using Calbryte 630 AM, represents transient cytosolic Ca^2^^+^ changes that occur immediately after peptide stimulation. The calcium flux was more pronounced in HEK293–GnRHR cells when exposed to a concentration of LA (100 nM) compared with HEK293–Vector cells. HS1002 (1 and 10 nM) increased calcium flux in HEK293–GnRHR and HEK293–Vectors cells, but only HS1002 (100 nM) increased calcium flux in HEK293–GnRHR cells (Figure 1H). Similar to the results of calcium flux assays, the activity of the CRE‐derived reporter gene was stimulated in HEK293–GnRHR cells by 100 nM LA. There was no substantial alteration in the CRE luciferase activity in HEK293–Vector cells. Interestingly, HS1002 concentration‐dependently enhanced the promoter binding activity mediated by cAMP. At 10 µM, HS1002 increased the twofold CRE‐luciferase activity compared with the control in HEK293‐GnRHR cells (Figure 1I). These data indicate that GnRHR serves as a promising molecular target for HS1002.

### 2.2 HS1002 Targets hTERT Expression and Telomerase Activity to Exhibit Antitumor Activity in LNCaP Cells

**FIGURE 2:**
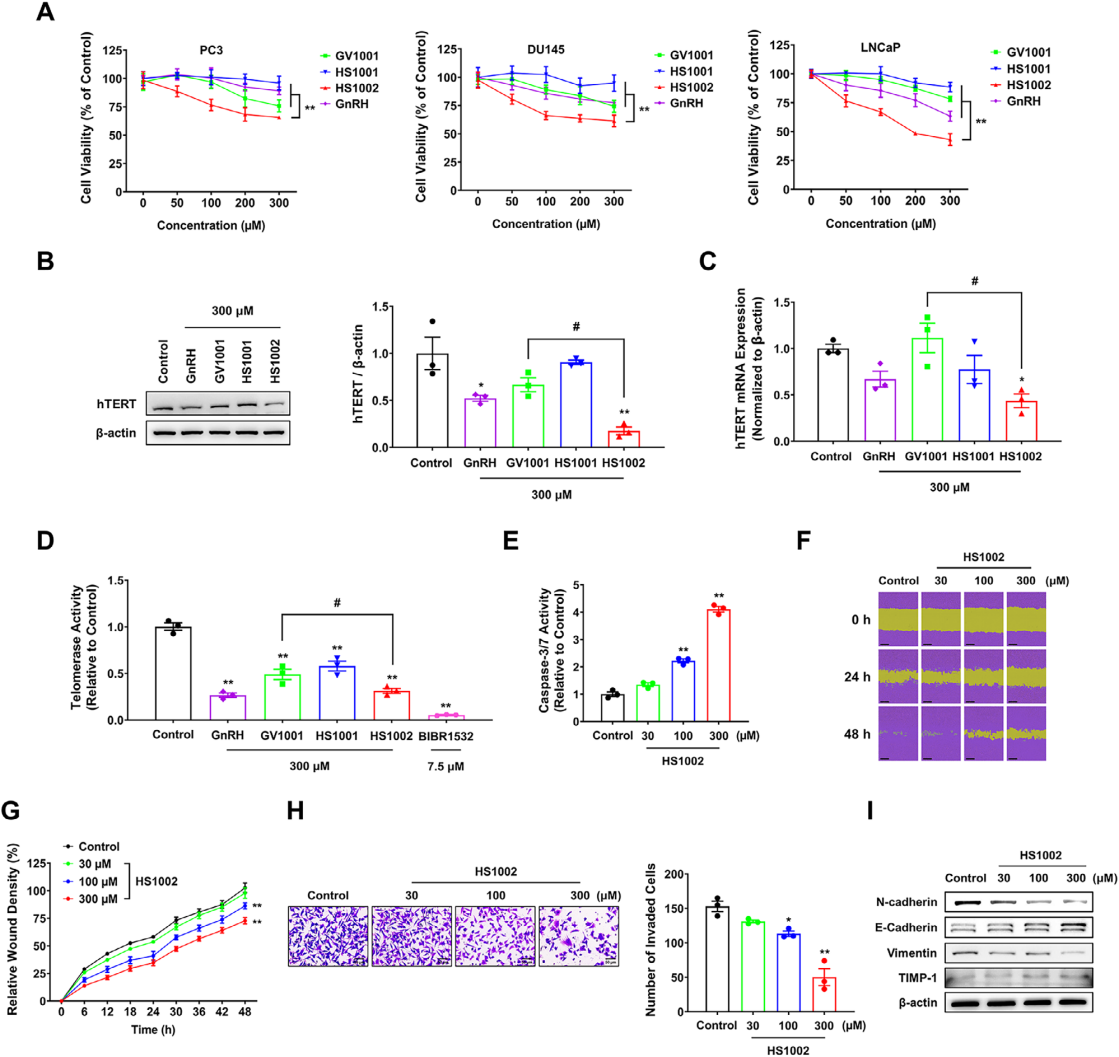
Effects of HS1002 on hTERT expression in prostate cancer cells. (A) Effect of different peptide derivatives on cell viability in prostate cancer cells. The values represent the means ± SD. **p < 0.01 between the two groups. (B) Western blot images and quantification of hTERT expression in peptide‐treated LNCaP cells. (C) hTERT mRNA expression levels were evaluated by qPCR. (D) Effect of different peptides (300 µM) on telomerase activity in LNCaP cells. BIBR1532 was used as a positive control. (E) Effect of HS1002 on caspase‐3/7 activity. (F and G) Wound healing assay of LNCaP cells treated with HS1002. Scale bar: 300 µm. (H) The invasive potential of LNCaP cells following HS1002 treatment was assessed using a Matrigel invasion assay. Scale bar: 50 µm. (I) Changes in levels of metastasis markers in LNCaP cells treated with HS1002. β‐Actin was used as a loading control. The band intensity was quantified using ImageJ software. All experiments were performed under 72 h treatment conditions; the wound healing assay was conducted over 48 h. The values represent the means ± SD. *p < 0.05 and **p < 0.01 versus control group; ^#^ *p* < 0.05 between two groups.

To investigate the relationship between hTERT and prostate cancer progression, hTERT expression was examined by immunohistochemistry (IHC) analysis in human prostate cancer tissue and compared with normal prostate tissue. The mean hTERT IHC score in prostate tumor tissues at Stages 3–4 and Gleason Grades 4–5 was markedly elevated compared with that in normal tissues and adjacent normal tissues (Figure S2A,B). Additionally, hTERT protein expression and telomerase activity were compared with examine their relationship in three prostate cancer cell lines. Both hTERT protein expression and telomerase activity were higher in PC3 cells than in LNCaP cells (Figure S2C,D). These data show that telomerase activity is directly correlated with hTERT expression and that hTERT upregulation has been linked to prostate cancer progression. MTT assay was performed on different prostate cancer cell lines for 72 h to evaluate the in vitro cytotoxic effects of the peptide derivatives. HS1001 was synthesized by combining hTERT:611–626 peptide segments (Glu–Ala–Arg–Pro–Ala–Leu–Leu–Ser–Thr–Arg–Leu–Arg), which are relevant for structural overlap and selectivity with the GnRH peptide, and Pro‐NHEt to enhance binding affinity. Treatment with HS1002 inhibited the viability of the prostate cancer cell lines, whereas GnRH and GV1001 marginally decreased cell viability (Figure 2A). Furthermore, HS1002 demonstrated the most substantial anticancer activity against prostate cancer cells in comparison with other types of cancer cells (Figure S2E). To determine whether HS1002 could affect telomerase activity in prostate cancer, we examined the changes in hTERT expression in LNCaP cells, which showed the most significant reduction in cell viability due to peptide derivatives. Interestingly, HS1002 significantly reduced hTERT protein and mRNA levels and suppressed telomerase activity in LNCaP cells relative to the control and GV1001 groups (Figure 2B–D). To determine whether the decrease in cell viability caused by HS1002 was a result of apoptosis, an Annexin V‐FITC and propidium iodide staining assay was conducted. After 72 h, the population of apoptotic cells treated with HS1002 exhibited a substantial increase compared with cells treated with GnRH or GV1001 (Figure S3A,B). It was further observed that Bax levels were dose‐dependently enhanced 72 h after exposing LNCaP cells to HS1002. In contrast, Bcl‐2 expression levels were reduced in LNCaP cells exposed to HS1002 (Figure S3C). Additionally, HS1002 increased caspase‐3/7 activity dose‐dependently (Figure 2E). To investigate whether nonapoptotic mechanisms contribute to HS1002‐induced cytotoxicity, we examined the involvement of autophagy. This was based on prior evidence linking GnRHR signaling to autophagy‐related cell death [17]. Upon HS1002 treatment, we observed increased formation of acidic vesicular organelles via acridine orange staining, indicative of autophagic activation (Figure S4A). Western blot analysis further revealed upregulation of autophagy‐related proteins, including Beclin‐1, Atg7, and LC3B‐II, along with decreased p62 levels (Figure S4B). Importantly, cotreatment with autophagy (3‐MA) or apoptosis (Z‐VAD–FMK) inhibitors significantly rescued cell viability, suggesting that autophagy also plays a key role in HS1002‐mediated cancer cell death (Figure S4C). The scratch wound assay showed that HS1002 treatment diminished the capacity for wound healing compared with the control group (Figure 2F,G). At 48 h postscratch, significant differences in relative wound healing were observed between the control cells and the cells treated with 100 or 300 µM HS1002. The matrigel invasion assay showed that HS1002 effectively inhibited the invasion of LNCaP cells (Figure 2H). Moreover, western blot analysis revealed that HS1002 caused a dose‐dependent increase in tissue inhibitor matrix metalloproteinase‐1 and E‐cadherin expression while decreasing N‐cadherin and vimentin expression (Figure 2I).

### 2.3 HS1002 Reduces hTERT Expression by Inhibiting c‐Myc and Associated Signaling Proteins in Prostate Cancer Cells

**FIGURE 3:**
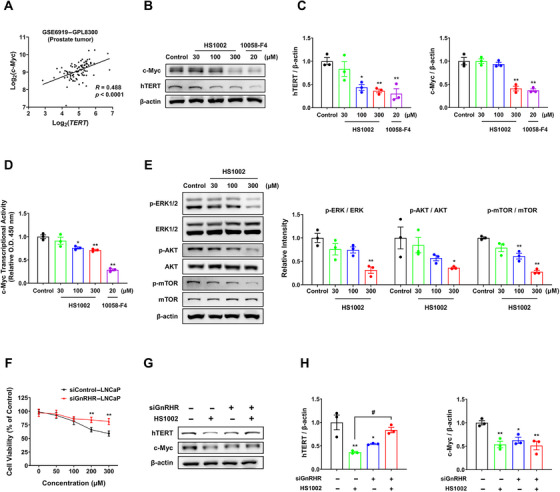
Suppression of hTERT‐regulating proteins by HS1002. (A) The relationship between c‐Myc and hTERT in prostate tumors. (B) Western blotting analysis of c‐Myc and hTERT expression after 72 h of treatment with HS1002 in LNCaP cells. (C) Band intensities of hTERT and c‐Myc in HS1002‐treated LNCaP cells were quantified using ImageJ software. (D) Effect of HS1002 treatment on c‐Myc transcriptional activity. (E) Changes in signaling protein expression (AKT, ERK1/2, and mTOR) after HS1002 treatment in LNCaP cells. (F) The effect of HS1002 treatment on proliferation in siControl and siGnRHR cells after 72 h. (G) Changes in hTERT and c‐Myc protein levels in siControl and siGnRHR LNCaP cells after HS1002 treatment. (H) Densitometric analysis of hTERT and c‐Myc in siControl and siGnRHR cells following HS1002 treatment. β‐Actin was used as a loading control. The values represent the means ± SD. *p < 0.05 and **p < 0.01 versus control group; ^#^ *p* < 0.05 between two groups.

To investigate the mechanism through which HS1002 modulates hTERT expression, we confirmed the expression of transcription factors and their upstream signaling proteins essential for hTERT regulation. First, analysis of the gene expression omnibus dataset showed that c‐Myc expression was highly correlated with hTERT expression in the transcripts of human prostate tumors (90 samples), indicating a possible relationship between the two genes in prostate cancer (Figure 3A). To confirm the connection between HS1002 and c‐Myc activity, the transcriptional activity of c‐Myc and its protein expression were measured after treatment with HS1002 or a c‐Myc inhibitor (10058‐F4). Both c‐Myc protein expression and transcriptional activity were significantly decreased in LNCaP cells treated with 300 µM HS1002 (Figure 3B–D). The expression levels of signaling proteins that regulate hTERT were evaluated using western blot analysis in LNCaP cells treated with HS1002. HS1002 significantly downregulated the expression of p‐ERK1/2, p‐AKT, and p‐mTOR (Figure 3E). These results indicate that HS1002 treatment resulted in a decrease in c‐Myc activity and phosphorylation of signaling proteins, causing decreased hTERT expression and activity. To further evaluate if AR (androgen receptor) status influenced the cellular response to HS1002, we extended our cytotoxicity assays to additional prostate cancer cell lines (DU145 and 22Rv1). HS1002 exhibited dose‐dependent cytotoxicity in both cell lines (Figure S5A). To assess whether these effects were consistently mediated through the similar pathway, we analyzed the expression levels of key signaling proteins. Notably, consistent modulation of these markers was observed in GnRHR‐expressing cell lines upon HS1002 treatment (Figure S5B,C). Furthermore, GnRHR knockdown was performed using siRNA to investigate whether the anticancer mechanism of HS1002 is mediated via GnRHR. The results showed that the basal proliferation rates of siGnRHR and siControl cells were similar. While HS1002 treatment for 72 h reduced proliferation in both cell types, the suppression of siGnRHR cell proliferation by HS1002 was much lower than that in siControl cells (Figure 3F). Moreover, the basal expression levels of hTERT and c‐Myc were lower in siGnRHR cells compared with siControl cells. Notably, HS1002‐induced downregulation of these proteins was observed only in siControl cells (Figure 3G,H). Because the antiproliferation effect of HS1002 in LNCaP cells was diminished by GnRHR reduction, the anticancer actions of HS1002 are likely to occur through GnRHR activation.

### 2.4 HS1002 Exhibits Potential Anticancer Effect by Reduction in Tumor Growth and hTERT Expression in a LNCaP Xenograft Model

**FIGURE 4:**
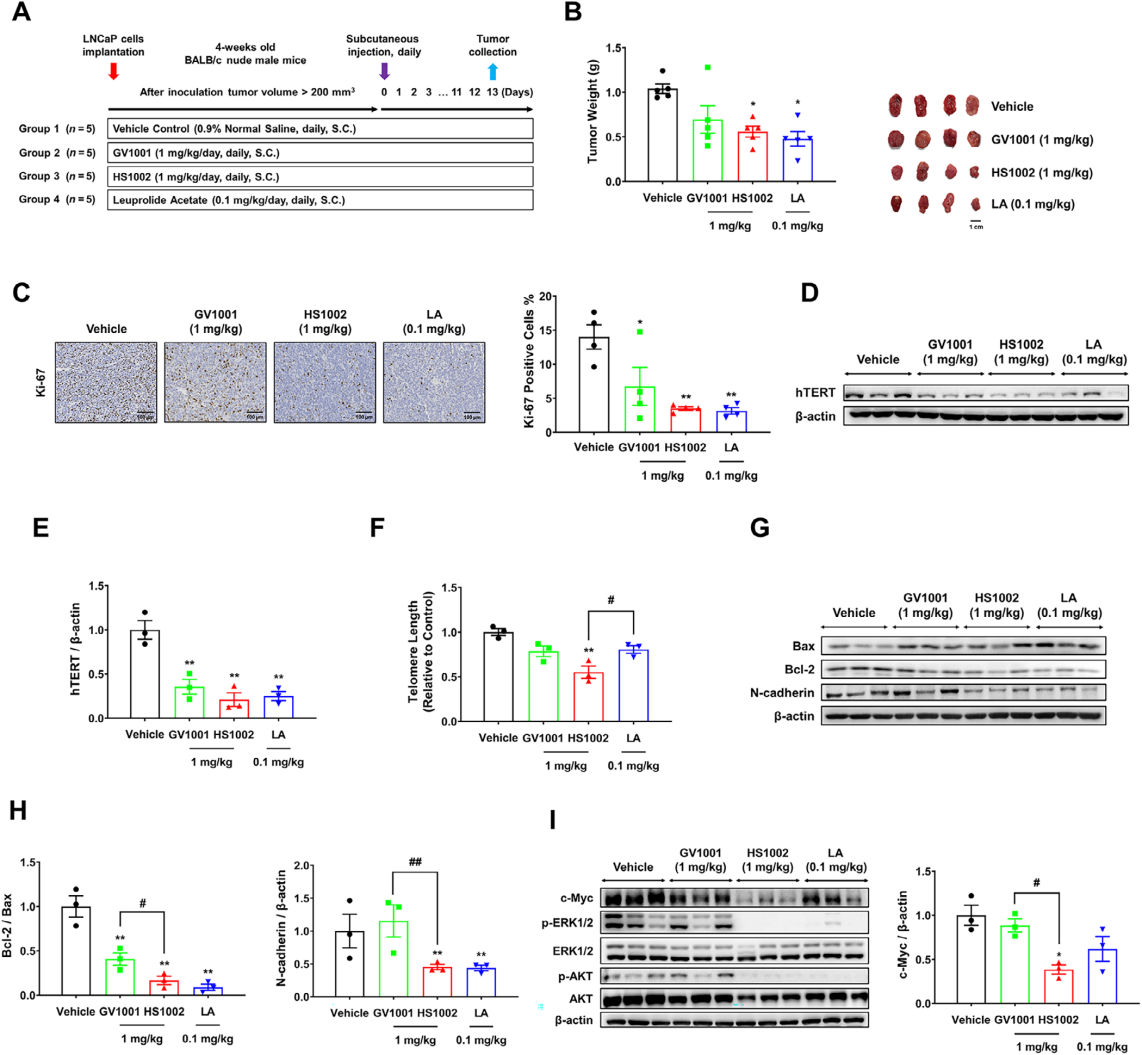
Inhibition of prostate tumor growth by HS1002 in a xenograft mouse model. (A) Schematic diagram of a xenograft mouse tumor model. (B) Tumor weight of LNCaP‐implanted mice injected with vehicle, GV1001, HS1002, or LA. (C) Ki‐67 expression in IHC‐stained tumor tissue sections. The proportion of Ki‐67‐positive cells was determined using ImageJ software. Scale bar: 100 µm. (D and E) Analysis of hTERT expression in LNCaP‐xenografted tumors by Western blot. (F) Telomere length measurement by qPCR. (G and H) Representative western images of Bax, Bcl‐2, and N‐cadherin in LNCaP‐xenografted tumors. (I) Change in the expression of c‐Myc and signaling proteins (ERK1/2, AKT) levels in LNCaP‐xenografted tumors. β‐Actin was used as a loading control. The values represent the means ± SD. *p < 0.05 and **p < 0.01 versus the vehicle group; ^#^ *p* < 0.05 and ^##^ *p* < 0.01 between two groups.

The effect of HS1002 on the tumor growth of LNCaP cells was evaluated in a BALB/c nude mouse xenograft model (Figure 4A). Subcutaneous injection of HS1002 or LA considerably suppressed tumor weight in LNCaP cell‐implanted nude mice, compared with the vehicle group (Figures 4B and S6A). No significant decrease in body weight was observed (Figure S6B). IHC examination revealed that Ki‐67 staining levels were reduced in the tumor tissues obtained from mice treated with HS1002 and LA compared with the vehicle group (Figure 4C). To examine the effect of HS1002 treatment on hTERT expression in tumor tissues, western blot analysis and telomere length assays were conducted. Western blot analysis indicated that the treatment with HS1002 led to a significant decrease in hTERT expression compared with the control group (Figure 4D,E). As shown in Figure 4F, the relative mean telomere length in the HS1002 group was less than that in the vehicle group. Based on these results, we concluded that HS1002 induces telomere shortening by suppressing hTERT expression in tumor tissues. A TUNEL assay demonstrated that HS1002 induced apoptotic cell death in LNCaP tumor tissues (Figure S6C,D). Moreover, as displayed in Figure 4G,H, HS1002 induced a distinct decrease in Bcl‐2/Bax and N‐cadherin in LNCaP xenografts compared with the vehicle group. In a similar in vitro study, HS1002 and LA treatments significantly suppressed the expression of c‐Myc, p‐AKT, and p‐ERK1/2. Interestingly, HS1002 decreased c‐Myc expression to a greater extent than LA (Figure 4I). Collectively, these data indicate that HS1002 treatment suppresses hTERT expression, which may be closely related to the decrease in levels of c‐Myc, ERK, and AKT in LNCaP tumor xenografts.

### 2.5 HS1002 Displays Immune Response Activation in Prostate Cancer Cells and MC38 Syngeneic Mouse Models

**FIGURE 5:**
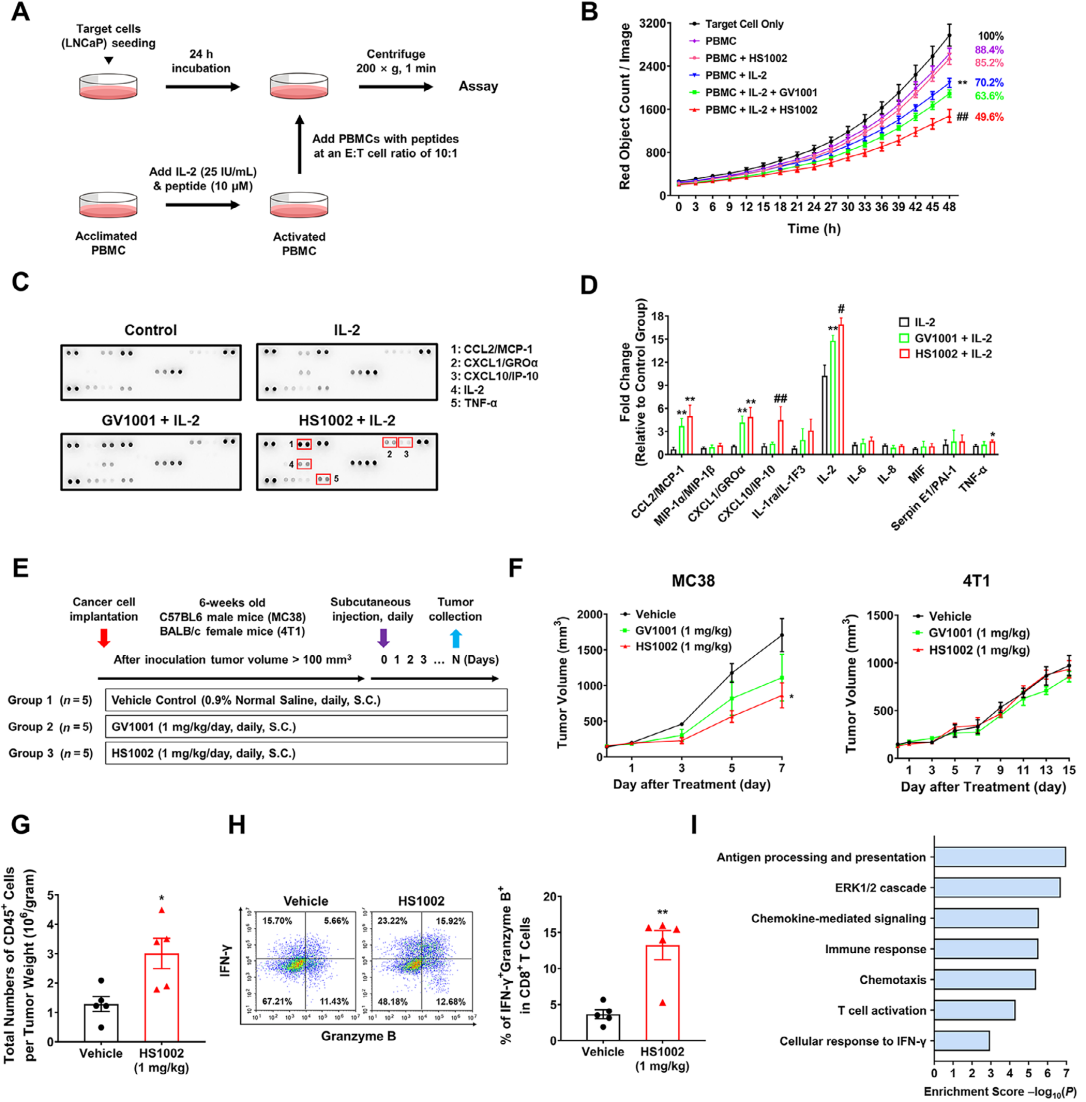
Enhancement of anticancer immunity by HS1002. (A) Schematic diagram of coculture of LNCaP cells with PBMC. (B) Cytotoxic activity of HS1002/IL‐2 or GV1001/IL‐2‐pretreated PBMC against LNCaP cells. **p < 0.01 versus PBMC group; ^##^ *p* < 0.01 versus PBMC/IL‐2 group. (C) Measurement of cytokines in the coculture supernatant of LNCaP cells with PBMC after peptide treatment. (D) Quantification of each dot intensity in the cytokine array. (E) Comparison of MC38 and 4T1 tumor growth inhibition by HS1002. Experimental scheme of a syngeneic mouse tumor model. (F) The growth curves of MC38 or 4T1‐implanted mice injected with vehicle, HS1002, or GV1001. (G) Effect of HS1002 on tumor‐infiltrated leukocytes in MC38 tumors. Absolute counts of CD45^+^ cells per gram tumor in a vehicle or HS1002‐treated MC38 tumor. (H) Percentages of granzyme B and IFN‐γ‐positive CD8^+^ cell populations in MC38 tumor‐bearing mice treated with vehicle or HS1002. (I) Biological process analysis was performed using DAVID on selected upregulated genes between vehicle‐ or HS1002‐treated MC38 tumors, using a cutoff of *p* < 0.05 and fold change >1.5. The values represent the means ± SD. *p < 0.05 and **p < 0.01 versus control group; ^#^ *p* < 0.05 and ^##^ *p* < 0.01 versus GV1001/IL‐2 group.

Confirming the antigen‐specific immune response of HS1002 is crucial for determining its capacity to trigger immunogenic cancer cell death. The ability of HS1002 to induce a specific cellular immune response was initially assessed using the IFN‐γ ELISpot assay. IL‐2 is an essential cytokine for activating and maintaining antigen‐specific T cells in an in vitro model to study the PBMC's immune response [18]. Therefore, PBMC were pre‐exposed to each peptide for 24 h, and the production of IFN‐γ in PBMC was measured. The HS1002 and GV1001 treatment groups exhibited significantly higher numbers of IFN‐γ spots in PBMCs compared with both the IL‐2 group and the PBMC control group (Figure S7A,B). To determine whether HS1002 activates immune cell responses against cancer cells, the effect of HS1002 on the cytotoxic activity of PBMC against LNCaP cells was investigated using appropriate effector/target ratios. PBMC were incubated separately with the peptides and IL‐2 (Figure 5A). After 24 h of incubation with HS1002 or GV1001, PBMC were added to LNCaP cells, and the cytotoxicity of PBMC on LNCaP cells was determined using Incucyte ZOOM analysis. The cytotoxicity of HS1002/IL‐2‐pretreated PBMC was significantly higher in LNCaP cells than that in IL‐2‐pretreated PBMC (Figures 5B and S8). Human cytokine array analysis was conducted to examine the differential secretion of cytokines by LNCaP/PBMC cocultures. No significant differences in cytokine levels were observed between cancer cells alone and IL‐2‐pretreated PBMC coculture supernatants in the human cytokine array. The HS1002 or GV1001/IL‐2‐pretreated PBMC coculture supernatants showed increased C–X–C motif chemokine ligand 1 (CXCL1), CXCL10, C–C motif chemokine ligand 2 (CCL2), tumor necrosis factor (TNF)‐α, and IL‐2 levels when compared with IL‐2‐pretreated PBMC coculture supernatants. Notably, IL‐2 and CXCL10 levels were increased in the HS1002/IL‐2‐pretreated PBMC coculture supernatants compared with those in the GV1001/IL‐2‐pretreated PBMC coculture supernatants (Figure 5C,D). These results suggest that HS1002 enhances PBMC‐mediated cytotoxicity and cytokine secretion against prostate cancer cells.

Previous research has demonstrated that tumor cells can present the hTERT antigen following hTERT peptide treatment, which increases the production of effector cells that specifically target tumor antigens [19]. Tumor growth inhibition was examined in the syngeneic mouse models to confirm the effect of HS1002 on anticancer immunity (Figure 5E). Interestingly, tumor volume and weight decreased when HS1002 or GV1001 were administered in the MC38 (hot tumor) model, but not in the 4T1 (cold tumor) model (Figure 5F). These results indicate that tumor immune microenvironment phenotypes may affect the antitumor effect of HS1002 in the syngeneic mouse model. CTLs are the key mediators of granzyme‐ and perforin‐mediated cancer cell destruction during immunotherapy. IFN‐γ also boosts the antitumor activities of other immune cells, suggesting that modulating CD8^+^ T cell activity can enhance antitumor immunity [20]. Initially, it was confirmed that HS1002 increased the quantity of TUNEL‐positive cells in the tumor tissue when compared with the vehicle group (Figure S9A). To evaluate the possibility of off‐target organ toxicity, histological assessments were conducted using hematoxylin and eosin (H&E) staining on the liver, kidney, spleen, and testes. No pathological abnormalities were observed in any of the examined organs throughout the in vivo study (Figure S9B). Next, we demonstrated that HS1002 increased the total number of CD45^+^ cells that infiltrate tumors in MC38 tumor tissue, as well as the levels of granzyme B and IFN‐γ production in CD8^+^ cells (Figure 5G,H). Furthermore, mouse cytokine array analysis was performed to identify cytokines that showed differential expression in the tumor microenvironment. The tumor treated with HS1002 exhibited elevated levels of CCL2, CCL3, CXCL9, IFN‐γ, interleukin‐1 receptor antagonist (IL‐1ra), and IL‐16 compared with the tumor treated with the vehicle (Figure S10A,B). Gene analysis was performed by profiling the tumor transcriptomes to assess the effect of HS1002 on immune response‐related pathways. A total of 49,196 genes were analyzed in tumor tissues, and genes with a fold change exceeding 1.5 were included for further analysis with statistical significance at *p* < 0.05. Gene ontology function analysis of these genes demonstrated a substantial association with immune response functions such as antigen processing and presentation, the ERK pathway, chemokine‐mediated signaling, chemotaxis, T cell activation, and cellular response to IFN‐γ (Figure 5I).

## 3 Discussion

Various GnRH peptide derivatives have been produced based on their structure–affinity relationships to enhance affinity and metabolic stability [21]. As a result, the NH_2_‐terminal domain (pGlu–His–Trp–Ser) of GnRH has been established as essential for receptor binding and activation, with Trp [3] identified as a crucial residue. The pyroglutamate at the N‐terminal provides some resistance to terminal peptidases [22], whereas the COOH‐terminal domain, specifically the Pro–Gly–NH_2_ sequence, plays a critical role in receptor binding. The COOH‐terminal tail can be replaced with an ethylamide, which also enhances metabolic stability [23]. In this study, novel peptides were constructed by introducing amino acid substitutions and cleavage around the Leu–Arg region of the hTERT:611–626 peptide. Initially, among the peptide derivatives evaluated, HS1002 showed the most substantial cytotoxicity against all prostate cancer cell lines expressing GnRHR. Since the most significant effects of HS1002 cytotoxicity were observed in the LNCaP cell line, LNCaP cells were further employed to confirm the effects of HS1002. Treatment with HS1002 led to a decrease in hTERT expression and telomerase activity in LNCaP cells, which was more significant than the reduction in hTERT caused by other peptide derivatives. This telomerase inhibition by HS1002 could be attributed to the inhibition of hTERT expression [24]. Additionally, apoptosis indices, such as caspase 3/7 activity and Bax expression, were induced by HS1002 in LNCaP cells. Previous studies have indicated that the proapoptotic and antiproliferative effects of GnRHR agonists are linked to the inhibition of hTERT mRNA expression and telomerase activity in steroid‐regulated cancers expressing GnRHR, likely mediated indirectly through a signaling pathway such as MAPK rather than directly affecting hTERT [25, 26]. Therefore, the antiproliferative and apoptotic effects of HS1002 may rely on its direct influence on GnRHR signaling. Subsequent suppression of hTERT expression in tumor cells leads to a reduction in telomere length, exerts antiproliferative effects, and initiates apoptosis [27].

In general, most cases of prostate cancer that are responsive to ADT progress to the stage of CRPC with a higher metastasis ability within about 2 years [28]. Castration resistance is mainly driven by persistent activation of AR signaling under androgen‐deprived conditions, which can result from AR splice variants, intratumoral androgen biosynthesis, or lncRNA‐dependent modulation of AR expression and function. This sustained AR activity has been linked to enhanced metastatic potential in advanced prostate cancer [29, 30, 31]. In this study, wound healing and the transwell migration assay provided evidence that HS1002 had potent antimetastatic properties against LNCaP cells, as observed by the reduction in the expression of metastasis proteins. Similarly, hTERT inhibition by BIBR1532 reduced cell metastasis by regulating several epithelial–mesenchymal transition markers in papillary thyroid carcinoma cells [32]. Although HS1002 exhibited clear antimetastatic activities in vitro, its therapeutic potential in clinically relevant metastatic environments, such as bone metastasis, has yet to be fully demonstrated. Further investigations employing advanced in vivo metastasis models are warranted to confirm and extend these observations.

Our findings indicate that HS1002 induces significant cytotoxicity in LNCaP cells, with apoptosis contributing to cell death. However, given that GnRH receptor‐mediated anticancer activity has also been associated with autophagy‐related cell death, we explored this mechanism [17]. The results revealed that HS1002 treatment enhances autophagic activity, as evidenced by the formation of acidic vesicular organelles and modulation of autophagy‐related proteins. Furthermore, the use of autophagy and apoptosis inhibitors partially rescued HS1002‐induced cytotoxicity, indicating that both mechanisms contribute to the HS1002's antitumor effects. These results align with previous studies demonstrating that GnRH overexpression can induce autophagy‐related apoptosis in pancreatic cancer cells through the downregulation of the Akt/ERK pathways [17].

Our study demonstrates that HS1002 exerts cytotoxic effects across various prostate cancer cell lines expressing GnRHR. Specifically, LNCaP cells, which are AR‐positive and castration‐sensitive, exhibited the most pronounced response to HS1002 treatment. In contrast, DU145 and PC3 cells, which are AR‐negative, and 22Rv1 cells, which are AR‐positive but castration‐resistant, were relatively less responsive [33, 34]. The AR is a crucial target that controls prostate cancer growth and regulates hTERT expression [35]. The induction of telomerase activity by androgens occurs through the binding of AR to the promoter region of the hTERT gene, resulting in increased transcription of hTERT in LNCaP cells that express functional AR [36]. A previous study has demonstrated that the hTERT peptide‐stimulated GnRHR/Gαs/cAMP pathway can simultaneously induce AR activation and Yes‐associated protein 1 (YAP1) inactivation via phosphorylation at Ser81 and Ser127, respectively [37]. YAP, the downstream effector of the Hippo pathway, directly associates with the hTERT promoter through TEAD transcription factors, thereby enhancing hTERT transcription and telomerase activity [38]. This potential crosstalk between the GnRHR and AR signaling may partly explain the variable cellular responses to HS1002 observed among prostate cancer cell lines with differing AR expression or activity levels. In fact, GnRH analogs show a more pronounced antiproliferative effect in AR‐positive prostate cancer cells compared with AR‐negative cells [39]. These observations suggest that while GnRHR‐mediated pathways play a central role in HS1002's anticancer activity, the presence of functional AR and the hormonal sensitivity of the cancer cells might influence the efficacy of the treatment.

The typical GnRHR downstream signaling pathway involves the activation of phospholipase C‐dependent calcium release through Gαq coupling by the GnRHR ligand, which in turn increases LH release [40]. In fact, a previous study found changes in intracellular signaling cascades induced by GnRH in GnRHR‐transfected HEK293 cells. Intracellular Ca^2+^ and cAMP accumulation showed dose‐dependent increases in GnRHR‐transfected HEK293 cells upon GnRH injection [41]. We demonstrated that HS1002 affects the GnRHR downstream signaling pathways, identifying HS1002 as a GnRHR ligand. GnRH analogs have distinctive effects in the pituitary gland (activation of the pituitary–gonadal axis) and in cancer cells (suppression of cancer cell proliferation and metastasis) [42]. These differences suggest that GnRHR can couple with different G proteins and intracellular signaling pathways depending on the cellular context [37].

Numerous transcription factors have been identified to be involved in controlling hTERT expression and telomerase activity [43]. The Myc protein plays a crucial role in activating telomerase by promoting hTERT transcription in neoplasms, such as prostate cancer [44]. The c‐Myc protein forms dimers with its binding partner MAX and interacts with the E‐box sequences found in the hTERT core promoter region. This interaction triggers hTERT transcription activation [45]. In our study, the analysis of a publicly available dataset also indicated that hTERT mRNA expression was found to have a positive correlation with the expression of c‐Myc mRNA in prostate cancers. A recent study also provided evidence that suppression of c‐Myc by baicalein was associated with hTERT inhibition in HL‐60 cells [46]. Therefore, c‐Myc could be a candidate modulator for the effects of HS1002 on hTERT expression. AKT, ERK, and mTOR signaling are also involved in the effect of hTERT activation [47, 48]. Phosphorylation of hTERT at Ser227 and Ser826 by AKT induces activation and facilitates the movement of hTERT into the nucleus [49, 50]. The regulatory role of mTOR in controlling telomerase activity has been established at both the translational and posttranslational levels. The mTOR inhibitor rapamycin was observed to reduce telomerase activity in prostate cancer cells [51]. Treatment with epidermal growth factor results in ERK phosphorylation, thereby initiating ERK signaling pathway activation in renal cell carcinoma cell lines. This activation subsequently leads to an upregulation of hTERT expression and increased telomerase activity [52]. Also, ERK binds directly to c‐Myc and phosphorylates it at Ser62, leading to the activation of c‐Myc and increased protein stability [53]. It was shown that HS1002 inhibited the phosphorylation of AKT, ERK1/2, and mTOR without significantly affecting basal protein levels. These results were consistent with those of other studies that have shown that GnRH analogs decrease the phosphorylation of AKT, ERK, and mTOR and c‐Myc expression.

HS1002 exhibited a more effective reduction in the protein levels of hTERT in tumor tissue. Both LA and HS1002 reduced the development of tumors and the quantity of cells expressing Ki‐67 in LNCaP‐derived tumor tissues in xenograft analyses. Moreover, HS1002 and LA treatments increased the TUNEL‐positive staining region, attributing tumor suppression of the GnRH analogs to apoptosis. Previous research has also identified that the hTERT promoter exhibits activation in response to a single dose of androgen in LNCaP cells. Significantly, in AR‐positive CWR22 xenograft tumors, androgen deprivation resulted in a reduction of hTERT expression, followed by a decrease in telomerase activity [36]. This supports the finding that HS1002, as a GnRH analog, reduces androgen levels and inhibits hTERT expression in tumor tissues, consequently reducing telomere length in the xenograft model. Although we confirmed that hTERT expression correlates with clinical features such as Gleason score and disease stage in patient samples, the in vivo validation was limited to the LNCaP xenograft model, which represents androgen‐sensitive prostate cancer. Since HS1002 primarily acts through hormone‐dependent GnRHR signaling, its therapeutic efficacy in vivo may be less pronounced in high‐grade or androgen‐independent tumors.

The hTERT:611–626 peptide demonstrates high promiscuity by binding to potential HLA‐Class I and II epitopes, suggesting the possibility of activating both CD4^+^ and CD8^+^ T cells, which are vital for tumor elimination. The HLA‐DP*0401/02‐restricted T cell clones specifically recognized the minimal sequence ALLTSRLRFI (615–624). On the other hand, the HLA‐DQ*04‐restricted T cell clones and the cytotoxic T cell clones recognized the overlapping minimal fragment TSRLRFIPK (618–626) [10]. HS1002 possesses this minimal hTERT fragment (RLRFIP) for major histocompatibility complex recognition, suggesting its potential as a cancer vaccine that shares similarities with the hTERT peptide:611–626. In the present study, HS1002 enhanced the efficacy of immune cell‐mediated tumor killing in PBMC coculture models. It is well known that IL‐2 plays an essential role in facilitating the proliferation and differentiation of mature T cells [54]. Cytotoxic CD8^+^ T cells activated by specific antigens effectively kill target cells by secreting perforin and granzymes [55]. However, the activation of CD8^+^ T cells in vitro requires either polyclonal stimulation through CD3/CD28 or antigen‐specific stimulation, both of which require IL‐2 [56, 57]. The cytotoxicity of PBMC against LNCaP cells was significantly higher in HS1002‐pretreated PBMC compared with no peptide or GV1001‐pretreated PBMC. Additionally, in the chemokine profiling of PBMC coculture supernatant, treatment with HS1002 increased the levels of secreted CCL2, CXCL10, IL‐2, and TNF‐α. CCL2, a chemokine with strong monocyte‐attracting properties, plays an important role in monocyte recruitment from the bloodstream to inflammation sites and tumor development [58]. The presence of the CCR2 receptor, which binds to the chemokine CCL2, has been demonstrated in a diverse range of cell types, such as NK cells, T lymphocytes, B lymphocytes, and neutrophils [59]. CXCL10, a chemokine that has an essential role in the recruitment of CTLs, is produced by immune cells, particularly macrophages and dendritic cells [60]. Elevated concentrations of CXCL10 have been correlated with the infiltration of effector CD8^+^ T cells in prostate cancer lesions [61].

Our data highlight enhanced CD8^+^ T cell responses in the responsive tumor model, supporting the notion that HS1002 can stimulate T cell‐mediated immunity. Notably, HS1002 contains a peptide sequence derived from hTERT, which has been previously reported to promote antitumor immune responses through mechanisms involving dendritic cell activation, regulatory T cell modulation, and cytotoxic T cell priming [62]. Based on these established immunological functions of hTERT‐derived peptides, it is conceivable that HS1002 may also affect other immune cell populations within the tumor microenvironment, such as dendritic cells, Tregs, and Myeloid‐derived suppressor cells. Although these effects remain to be fully elucidated, they may partly explain the selective efficacy of HS1002 in immunologically active tumor models.

Although high micromolar concentrations were required to induce detectable effects in cell‐based assays, daily subcutaneous administration at a comparatively lower systemic exposure effectively suppressed tumor growth and modulated GnRHR‐related signaling in vivo. This discrepancy may be attributable to the absence of systemic hormonal regulation under in vitro conditions, while additional GnRHR‐mediated effects under physiological conditions could modulate the in vivo responses [63, 64, 65]. The in vitro studies aimed to demonstrate the receptor‐dependent signaling mechanisms of HS1002 rather than to model physiological exposure, whereas distinct mechanisms in vivo, including pituitary‐mediated hormonal suppression and repeated subcutaneous dosing, could result in more sustained signaling responses. These findings emphasize the need for comprehensive in vivo evaluation, including pharmacokinetic, maximum tolerated dose, and organ distribution studies, to better support future preclinical optimization and translational development of HS1002. Peptides are often limited by several challenges, including their poor oral bioavailability, short half‐life, low cell membrane permeability, rapid enzymatic degradation in vivo, immunogenicity, and high renal clearance, along with stability and delivery issues. Therefore, managing these limitations is crucial to increasing the overall therapeutic effectiveness of novel peptides as an anticancer agent. To improve peptide stability such as in HS1002, various chemical modifications can be employed. Strategies such as PEGylation or peptide cyclization protect the peptide structure from enzymatic degradation, leading to a prolongation in its in vivo activity. Additionally, extension of half‐life can be attained by making modifications such as Fc fusion to the peptide structure, which can delay its renal clearance [66, 67]. By leveraging these techniques, peptide‐based therapies could overcome existing limitations, leading to an effective cancer treatment in the future.

## 4 Conclusion

**FIGURE 6:**
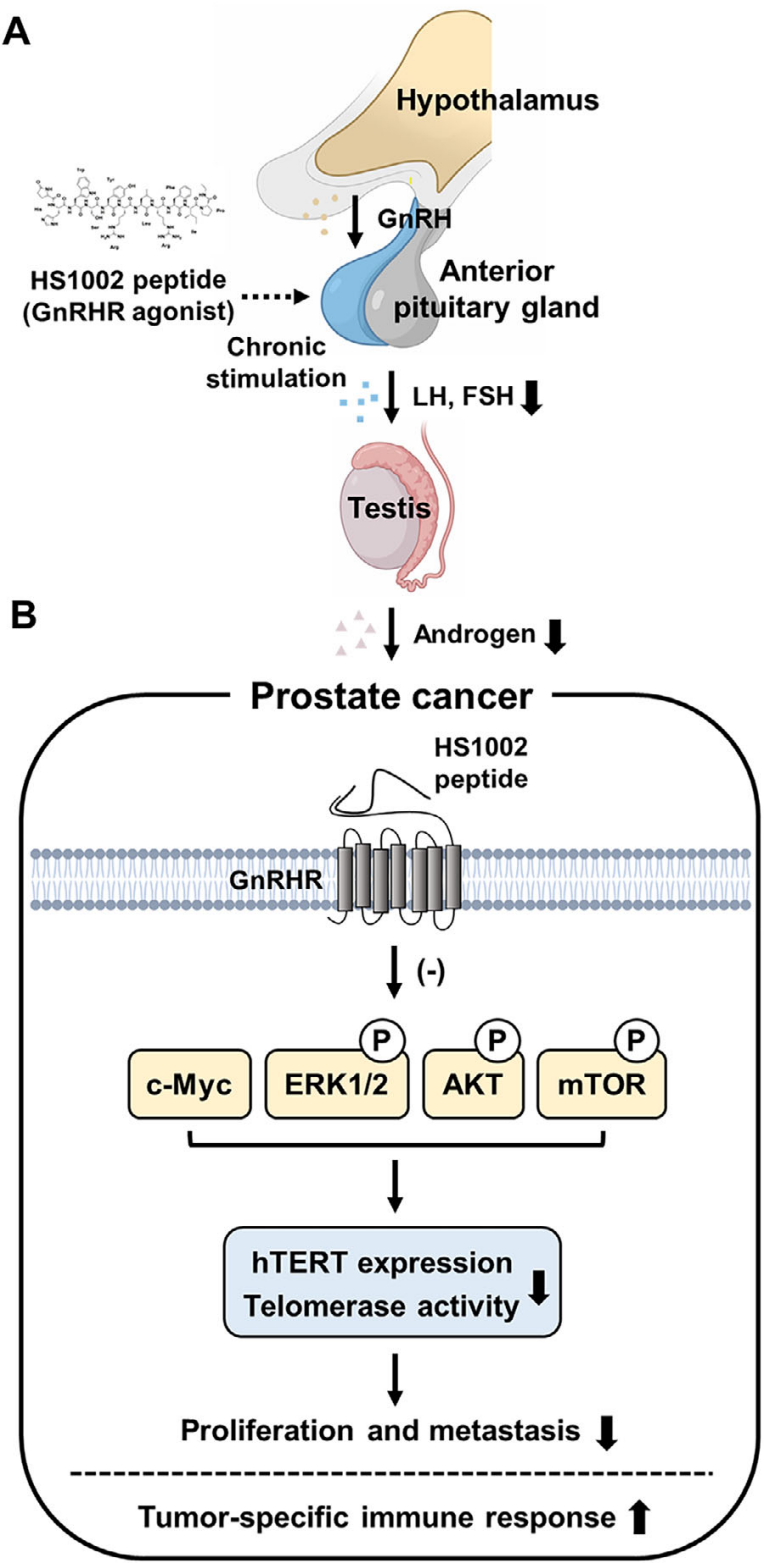
Schematic illustration of the anticancer mechanism of HS1002. The mechanism of HS1002 can be attributed to: (A) the reduction of serum testosterone levels by desensitization of GnRHR. (B) The downregulation of hTERT expression and telomerase activity and the enhancement of anticancer immunity.

Taken together, these data show that HS1002 diminishes prostate tumor growth by displaying multifaceted effects (Figure 6A,B). The effects of HS1002 can be attributed to: (a) the downregulation of c‐Myc and ERK, leading to the inhibition of hTERT expression and telomerase activity in prostate cancer cells; (b) the GnRHR desensitization and eventually the reduction of serum testosterone levels in male mice; and/or (c) the anticancer immune response that regulates lymphocyte infiltration and CD8^+^ T cell function in an in vivo syngeneic mouse. Consequently, it is suggested that HS1002 shows therapeutic potential in prostate cancer by exhibiting a mechanism of action involving the inhibition of prostate cancer cell growth and the enhancement of antitumor immunity.

## 5 Materials and Methods

### 5.1 Molecular Docking Study

To build a docking model of peptide HS1002 complexed with GnRHR (human), the Bioluminate (v3.6) program included in the Schrodinger molecular modeling S/W package was utilized for peptide docking. The preparation of the peptide and target receptor for docking analysis was carried out using the peptide docking module. The homology model of the target receptor (Uniprot = P01148) was obtained from the AlphaFold website (https://alphafold.ebi.ac.uk/). To prepare the structure of the target protein for docking, the receptor structure was briefly minimized at pH 7.0 utilizing the protein preparation module. The peptide ligands were sketched in the peptide docking panel, and the docking site was assigned by selecting the amino acid residues. The select residues were Gly26, Asn27, Asn102^2.65^, Thr198, Asn212^5.39^, Asn298, and Asp302^7.32^. A minimum of 10 docking poses were generated for each peptide, and docking scores were calculated using the MM‐GBSA method to estimate the binding affinity. Although the N‐terminal residue of the peptide used in docking was a noncanonical pyroglutamic acid (pGlu), it was docked as a canonical glutamic acid to be compatible with the forcefields (OPLS4 and OPLS5) for the docking algorithm. Likewise, the C‐terminal end was docked after being converted into natural amino acids. After the docking run, both peptide terminals were remodified into the original pGlu, C‐terminal amide, or ethyl amide capping, and then briefly energy‐minimized to construct the final models. These curation procedures were conducted using Cresset Flare v8.0.0.

### 5.2 Telomerase Activity and Telomere Length Assay

Telomerase activity was assessed using a telomerase activity quantification qPCR assay kit (ScienCell, Carlsbad, CA, USA). Cells were treated with each peptide (300 µM) for 72 h. Cellular proteins were isolated using cell lysis buffer with PMSF (0.1 M) and β‐mercaptoethanol. qPCR was conducted in a final volume of 20 µL using a posttelomerase reaction sample (1 µL), TPS (2 µL), 2 × qPCR FastStart Essential Green Master Mix (10 µL; Roche Diagnostics International), and nuclease‐free water (7 µL). The ∆Cq value was determined using the following equation: ∆Cq = Cq (positive control) − Cq (treatment). The formula (2^−∆Cq^) was used to calculate the relative telomerase activity. The relative telomere length of the tumor tissues was measured using a relative human telomere length quantification RT‐qPCR assay kit (Cat. 8908; ScienCell). Genomic DNA was extracted from the tumor tissues and employed as a template for qRT‐PCR. The data were evaluated using the comparative quantification cycle value (∆∆Cq) method.

### 5.3 Animal Study

All mice were obtained from Jung Ang Lab Animal Inc. (Seoul, South Korea). They were raised in a 12 h environmental light/dark cycle with controlled temperature (22 ± 2°C) conditions. The experimental protocol received approval from the Sungkyunkwan University Institutional Animal Care and Use Committee (SKKUIACUC2021‐06‐26‐1, SKKUIACUC2021‐06‐27‐1 and SKKUIACUC2023‐01‐07‐1). To establish tumors, LNCaP (1 × 10^7^), MC38 (2 × 10^5^), and 4T1 (1 × 10^6^) cells suspended in serum‐free medium containing 50% Matrigel were injected to the right flank of the mice subcutaneously. Tumor volumes were determined by measuring them with a caliper and applying the conventional equation: *V* (mm^3^) = 0.52 (*ab* ^2^) (*a* = length, *b* = width). After the tumor reached 100–200 mm^3^, the mice were divided into different groups randomly and injected with the vehicle or each peptide subcutaneously.

### 5.4 Tumor Digestion and Flow Cytometry

A mouse tumor dissociation kit (Cat. 130‐096‐730; Miltenyi Biotec, Bergisch Gladbach, Germany) was employed for tumor digestion. The tumor was cut into small pieces using RPMI 1640, which contains enzymes D, R, and A. The red blood cells were eliminated by using RBC lysis buffer (Cat. 420301; BioLegend, San Diego, CA, USA). To analyze intracellular cytokines, the cell suspension was treated with a cell stimulation cocktail (Cat. 00‐4975‐93; Invitrogen, eBioscience) for 5 h and subjected to fixation and permeabilization buffer (Cat. 88‐8824‐00; Thermo Fisher Scientific). After staining, the samples were analyzed using NovoCyte (ACEA Biosciences, San Diego, CA, USA).

### 5.5 Statistical Analysis

All the data are reported as the mean ± standard deviation (SD) of a minimum of three independent experiments. Statistical significance was analyzed using one‐way analysis of variance, followed by Tukey's posthoc comparison test. Analyses were conducted using GraphPad Prism Software (version 5.0; GraphPad Software, CA, USA). Statistical significance was set at a *p* value < 0.05, which indicated statistical significance.

More detailed materials and methods information is available in Supporting Information.

## Author Contributions

Hyung Sik Kim and Jae Hyeon Park designed the study. Hyung Sik Kim and Hyun‐Ju Park supervised the study. Jae Hyeon Park and Joo Chan Lee performed the experiments. Jae Hyeon Park, Swati Sharma, Chunxue Jiang, Haeun Lee, and Hyun‐Ju Park analyzed the data. Jae Hyeon Park, Joo Chan Lee, and Hyun‐Ju Park prepared the manuscript. All the authors have read and approved the final manuscript.

## Funding

This research was funded by the Ministry of Education (MOE, Korea) and National Research Foundation of Korea (NRF) (grant numbers 2018M3A9C8021792 and RS‐2024‐00356179).

## Ethics Statement

All applicable international, national, and institutional guidelines for animal care and use were adhered to. All animal procedures were performed in accordance with the ethical standards of the institution where the studies were conducted.

## Conflicts of Interest

The authors declare no conflicts of interest.

## Supporting information




**Figure S1**: In vivo administration of HS1002 affects the weight of seminal vesicles. (A) Experimental design. The seminal vesicles and testes were obtained from BALB/c mice following 10 days of administration with vehicle or HS1002 (1 mg/kg). (B) Representative images of seminal vesicles and testes.
**Figure S2**: The upregulation of hTERT is positively associated with the severity of prostate cancer. (A) Representative IHC images of tissue microarray stained for hTERT. The IHC analysis was conducted on samples of prostate cancer and normal prostate tissue to identify changes in hTERT expression. Scale bar: 200 µm. (B) hTERT immunostaining scores of normal prostate and prostate cancer tissue samples in the different tumor stages and Gleason grade. The values represent the means ± SD. *p < 0.05 and **p < 0.01 vs. the normal tissue group. Normal adjacent tissues; NAT. (C) hTERT expression levels in prostate cancer cell lines. β‐actin was used as a loading control. The band intensity was quantified using ImageJ software. (D) Relative telomerase activity in prostate cancer cell lines. (E) Effect of HS1002 on cell viability against different cancer cell lines. *p < 0.05 and **p < 0.01 vs. control group. The values represent the means ± SD.
**Figure S3**: HS1002 induces apoptosis in LNCaP cells. (A and B) Flow cytometric analysis of the percentage of apoptotic cells in peptide‐treated LNCaP cells. (C) Changes in apoptotic protein expression after 72 h of HS1002 treatment. β‐Actin was used as a loading control. The values represent the means ± SD. **p < 0.01 vs. control group. ^#^ *p* < 0.05 and ^##^ *p* < 0.01 vs. between two groups.
**Figure S4**: Autophagy contributes to HS1002‐induced cytotoxicity in LNCaP cells. (A) Formation of acidic vesicular organelles (AVOs) in LNCaP cells treated with HS1002, visualized by acridine orange staining. green: cytoplasm; red: AVOs; yellow: merged signal. Scale bar: 30 µm. (B) Western blot analysis of autophagy‐related proteins (Beclin‐1, Atg7, LC3B, and p62) in HS1002‐treated cells. β‐actin was used as a loading control. (C) Cell viability of LNCaP cells cotreated with the apoptosis inhibitor Z‐VAD‐FMK or the autophagy inhibitor 3‐MA in the presence of HS1002. The values represent the means ± SD. *p < 0.05 and **p < 0.01 vs. control group. ^##^ *p* < 0.01 vs. HS1002‐only treated group.
**Figure S5**: Effect of HS1002 on cell viability and hTERT‐related signaling in 22Rv1 and DU145 prostate cancer cell lines. (A) Cell viability of 22Rv1 and DU145 cells following 72 h of HS1002 treatment. (B) Western blot analysis of hTERT‐related signaling proteins (c‐Myc, hTERT, AKT, ERK1/2, and mTOR) in 22Rv1 and DU145 cells treated with HS1002. β‐Actin was used as a loading control. The values represent the means ± SD. *p < 0.05 and **p < 0.01 vs. control group.
**Figure S6**: HS1002‐induced tumor suppression and apoptosis in LNCaP xenograft models. (A) Representative images, (B) Body weight change of tumor‐implanted mice treated with vehicle, GV1001, HS1002, or LA. (C) Representative images of the TUNEL assay in xenografted LNCaP tumor tissue. Scale bar: 50 µm. (D) TUNEL‐positive cells were quantified using ImageJ software. The values represent the means ± SD. *p < 0.05 vs. the vehicle group.
**Figure S7**: Evaluating the immunogenicity of peptides by IFN‐γ ELISpot assay. (A) Representative IFN‐γ ELISpot images of PBMC exposed to GV1001 or HS1002 peptides (B) Quantification of the number of spots observed in the ELISpot assay. The values represent the means ± SD. **p < 0.01 vs. the PBMC group; ^#^ *p *< 0.05 and ^##^ *p* < 0.05 vs. the IL‐2 only group.
**Figure S8**: Cytotoxic effects of HS1002/IL‐2‐ or GV1001/IL‐2‐pretreated PBMCs on LNCaP cells. LNCaP cells expressing NucLight Red were cocultured with HS1002/IL‐2‐ or GV1001/IL‐2‐treated PBMCs for 48 h. Red fluorescence indicates viable LNCaP cells. Scale bar: 400 µm.
**Figure S9**: Evaluation of HS1002‐induced apoptosis and off‐target organ toxicity in MC38 syngeneic mouse models. (A) TUNEL assay for detection of apoptosis in tumor tissue sections. TUNEL‐positive cells were determined by ImageJ software. Scale bar: 100 µm. (B) Representation images of organ sections stained with H&E. The values represent the means ± SD. Scale bar: 200 µm in 40×, 100 µm in 200×. **p < 0.01 vs. the vehicle group.
**Figure S10**: Cytokine profiling in MC38 tumor tissues following HS1002 treatment. (A, B) Measurement of cytokines in vehicle or HS1002‐treated MC38 tumor tissues by cytokine array. The values represent the means ± SD. *p < 0.05 vs. the vehicle group.

## Data Availability

The datasets used and/or analyzed during the current study are available from the corresponding author upon reasonable request.
