# Supplementary material for: A Novel Peptide, HS1002, Enhances Antitumor Activity via Dual Targeting of the GnRH Receptor and Human Telomerase Reverse Transcriptase in Prostate Cancer Cells
Source: MedComm (2020). 2026 Feb 15;7(3):e70630. doi: 10.1002/mco2.70630 (PMC12906662; doi:10.1002/mco2.70630)
Supplement: Supplementary file 1 — Figure S1: In vivo administration of HS1002 affects the weight of seminal vesicles. (A) Experimental design. The seminal vesicles and testes were obtained from BALB/c mice following 10 days of administration with vehicle or HS1002 (1 mg/kg). (B) Representative images of seminal vesicles and testes. Figure S2: The upregulation of hTERT is positively associated with the severity of prostate cancer. (A) Representative IHC images of tissue microarray stained for hTERT. The IHC analysis was conducted on samples of prostate cancer and normal prostate tissue to identify changes in hTERT expression. Scale bar: 200 µm. (B) hTERT immunostaining scores of normal prostate and prostate cancer tissue samples in the different tumor stages and Gleason grade. The values represent the means ± SD. *p < 0.05 and **p < 0.01 vs. the normal tissue group. Normal adjacent tissues; NAT. (C) hTERT expression levels in prostate cancer cell lines. β‐actin was used as a loading control. The band intensity was quantified using ImageJ software. (D) Relative telomerase activity in prostate cancer cell lines. (E) Effect of HS1002 on cell viability against different cancer cell lines. *p < 0.05 and **p < 0.01 vs. control group. The values represent the means ± SD. Figure S3: HS1002 induces apoptosis in LNCaP cells. (A and B) Flow cytometric analysis of the percentage of apoptotic cells in peptide‐treated LNCaP cells. (C) Changes in apoptotic protein expression after 72 h of HS1002 treatment. β‐Actin was used as a loading control. The values represent the means ± SD. **p < 0.01 vs. control group. # p < 0.05 and ## p < 0.01 vs. between two groups. Figure S4: Autophagy contributes to HS1002‐induced cytotoxicity in LNCaP cells. (A) Formation of acidic vesicular organelles (AVOs) in LNCaP cells treated with HS1002, visualized by acridine orange staining. green: cytoplasm; red: AVOs; yellow: merged signal. Scale bar: 30 µm. (B) Western blot analysis of autophagy‐related proteins (Beclin‐1, Atg7, LC3B, and [file MCO2-7-e70630-s001.docx]

Supporting Information for

**“A novel peptide, HS1002, enhances antitumor activity via dual targeting of the GnRH receptor and human telomerase reverse transcriptase (hTERT) in prostate cancer cells”**

Running title: HS1002 as a dual-targeting peptide for cancer

Jae Hyeon Park, Joo Chan Lee, Swati Sharma, Chunxue Jiang, Haeun Lee, Hyun-Ju Park*, Hyung Sik Kim*

School of Pharmacy, Sungkyunkwan University, Suwon 16419, Republic of Korea

*Corresponding authors: hkims@skku.edu (Hyung Sik Kim); hyunju85@skku.edu (Hyun-Ju Park)

**Supplementary Figures**


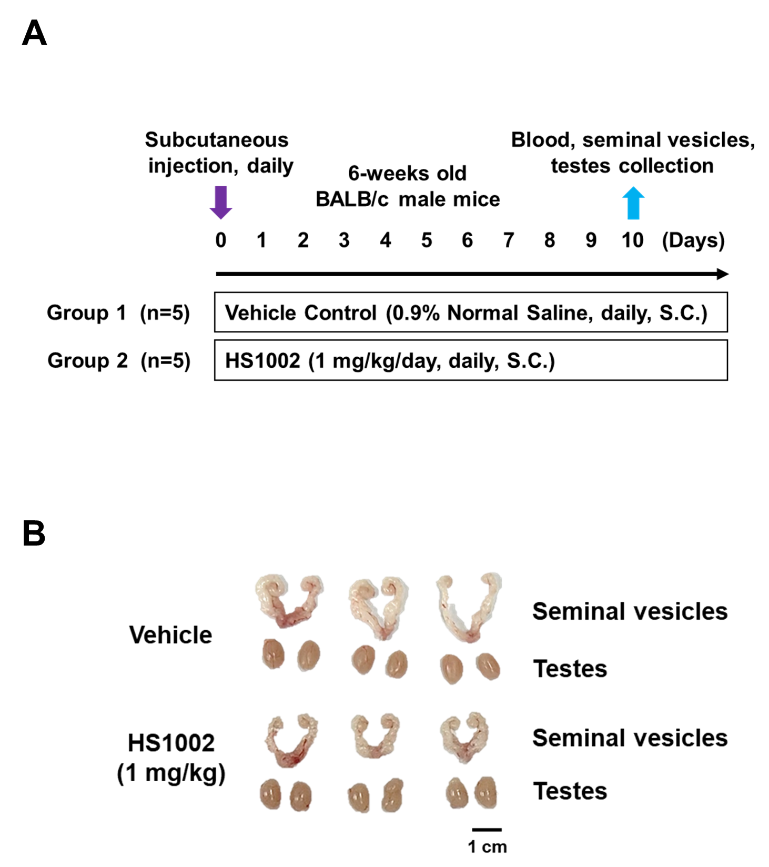


**FIGURE S1 In vivo administration of HS1002 affects the weight of seminal vesicles.** (A) Experimental design. The seminal vesicles and testes were obtained from BALB/c mice following 10 days of administration with vehicle or HS1002 (1 mg/kg). (B) Representative images of seminal vesicles and testes.


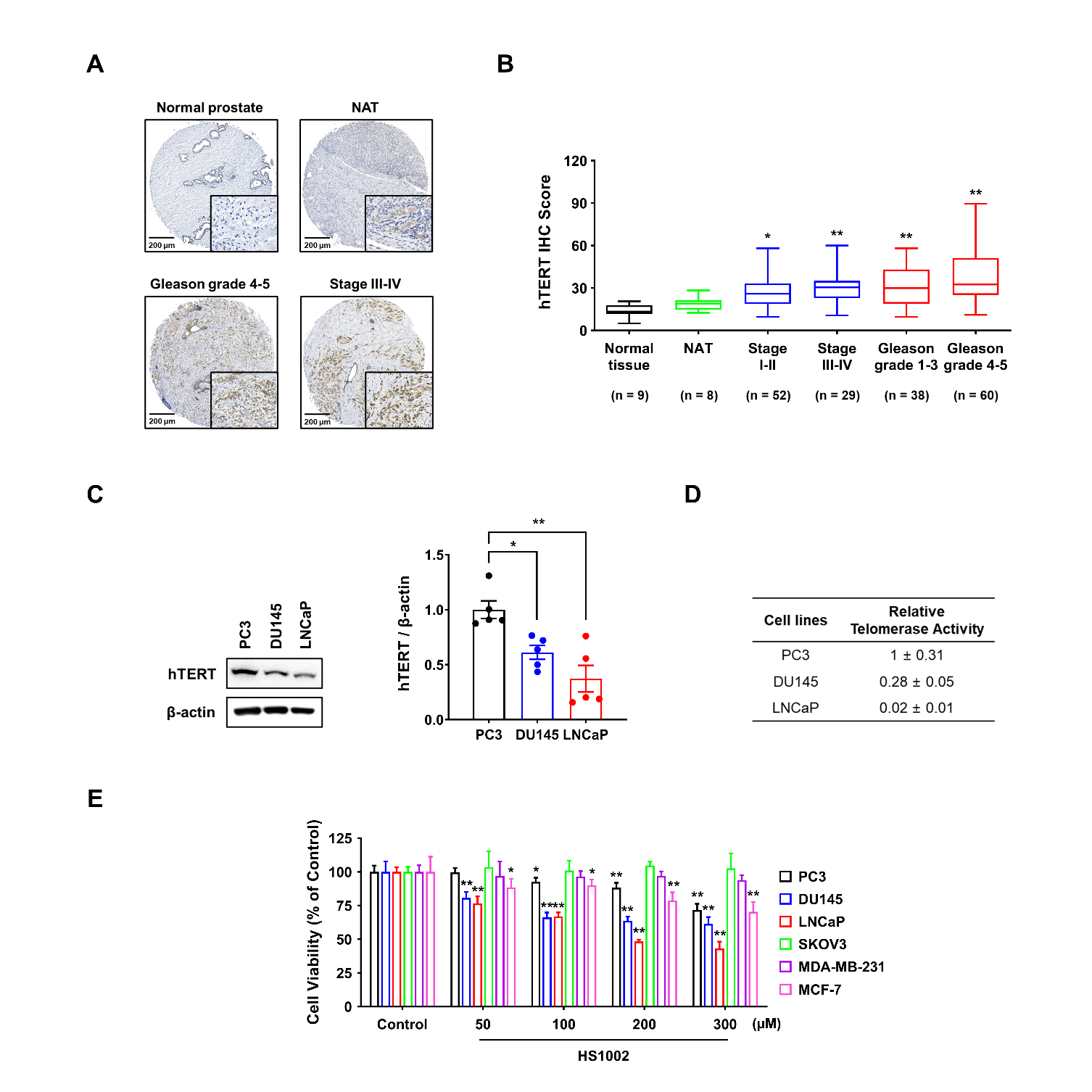


**FIGURE S2 The upregulation of hTERT is positively associated with the severity of prostate cancer.** (A) Representative IHC images of tissue microarray stained for hTERT. The IHC analysis was conducted on samples of prostate cancer and normal prostate tissue to identify changes in hTERT expression. Scale bar: 200 µm. (B) hTERT immunostaining scores of normal prostate and prostate cancer tissue samples in the different tumor stages and Gleason grade. The values represent the means ± SD. **p* < 0.05 and ***p* < 0.01 vs. the normal tissue group. Normal adjacent tissues; NAT. (C) hTERT expression levels in prostate cancer cell lines. β-actin was used as a loading control. The band intensity was quantified using ImageJ software. (D) Relative telomerase activity in prostate cancer cell lines. (E) Effect of HS1002 on cell viability against different cancer cell lines. **p* < 0.05 and ***p* < 0.01 vs. control group. The values represent the means ± SD.


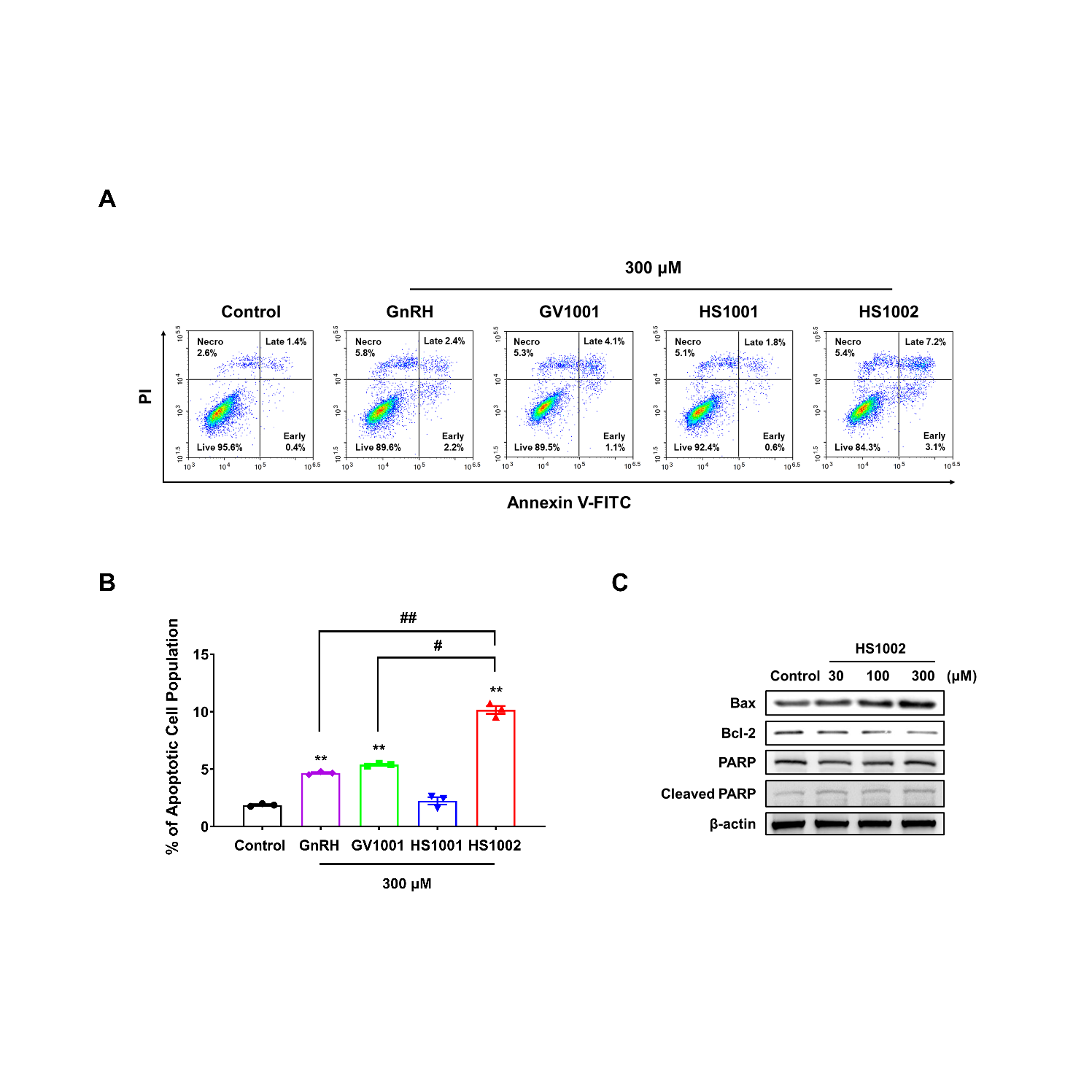


**FIGURE S3 HS1002 induces apoptosis in LNCaP cells.** (A, B) Flow cytometric analysis of the percentage of apoptotic cells in peptide-treated LNCaP cells. (C) Changes in apoptotic protein expression after 72 h of HS1002 treatment. β-actin was used as a loading control. The values represent the means ± SD. ***p* < 0.01 vs. control group. ^#^*p* < 0.05 and ^##^*p* < 0.01 vs. between two groups.


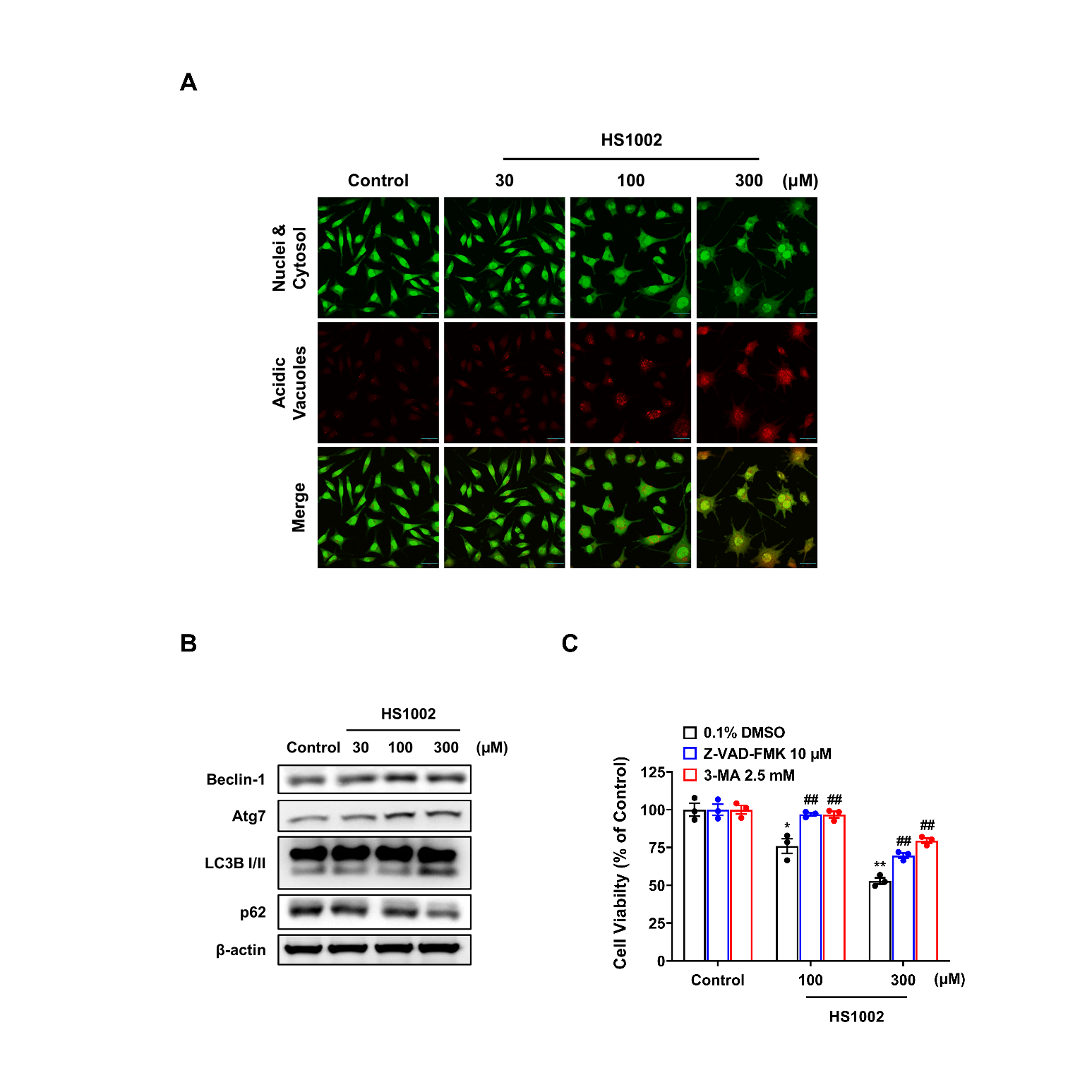


**FIGURE S4. Autophagy contributes to HS1002-induced cytotoxicity in LNCaP cells.** (A) Formation of acidic vesicular organelles (AVOs) in LNCaP cells treated with HS1002, visualized by acridine orange staining. green: cytoplasm; red: AVOs; yellow: merged signal. Scale bar: 30 µm. (B) Western blot analysis of autophagy-related proteins (Beclin-1, Atg7, LC3B, and p62) in HS1002-treated cells. β-actin was used as a loading control. (C) Cell viability of LNCaP cells co-treated with the apoptosis inhibitor Z-VAD-FMK or the autophagy inhibitor 3-MA in the presence of HS1002. The values represent the means ± SD. **p* < 0.05 and ***p* < 0.01 vs. control group. ^##^*p* < 0.01 vs. HS1002-only treated group.


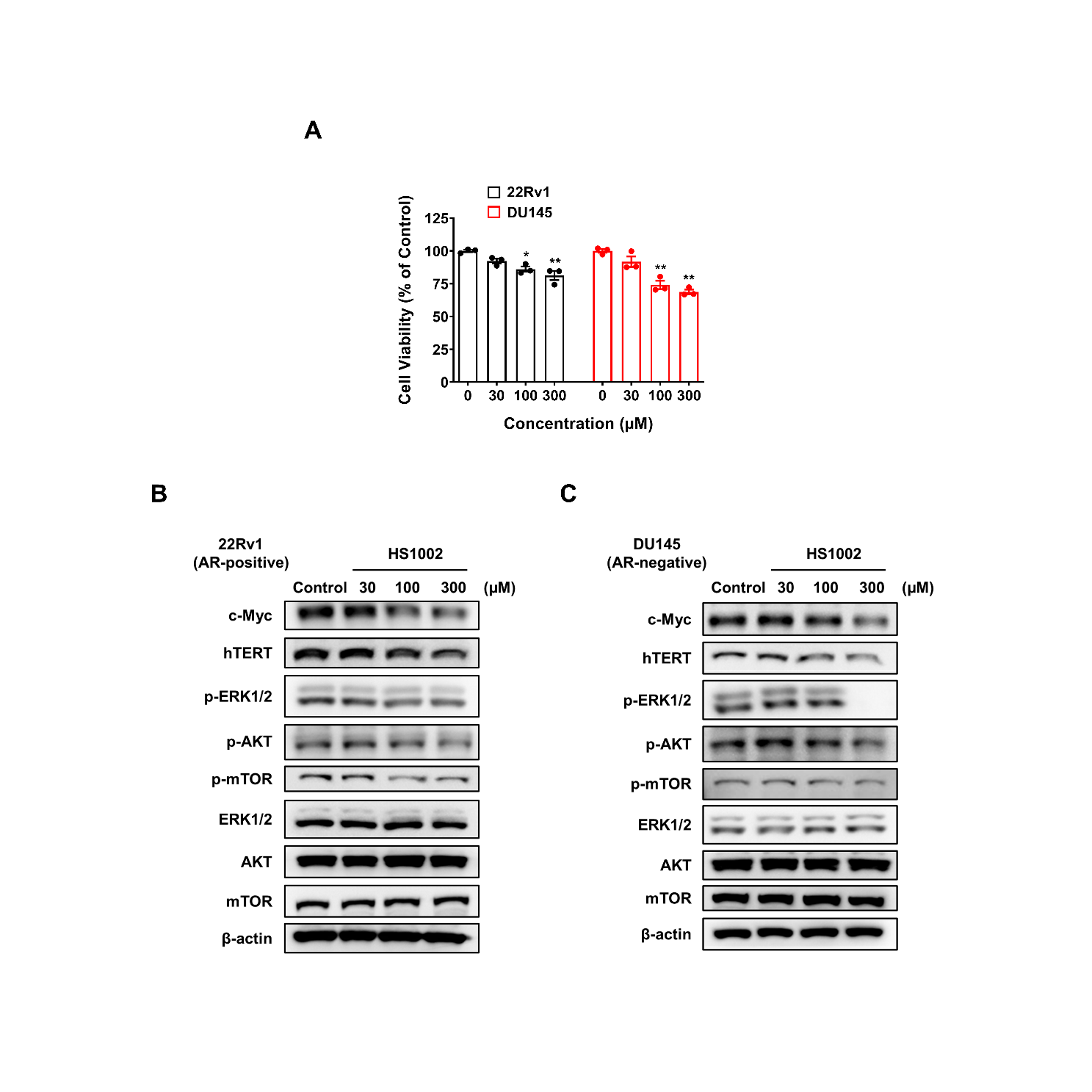


**FIGURE S5** **Effect of HS1002 on cell viability and hTERT-related signaling in 22Rv1 and DU145 prostate cancer cell lines.** (A) Cell viability of 22Rv1 and DU145 cells following 72 h of HS1002 treatment. (B) Western blot analysis of hTERT-related signaling proteins (c-Myc, hTERT, AKT, ERK1/2, and mTOR) in 22Rv1 and DU145 cells treated with HS1002. β-actin was used as a loading control. The values represent the means ± SD. **p* < 0.05 and ***p* < 0.01 vs. control group.


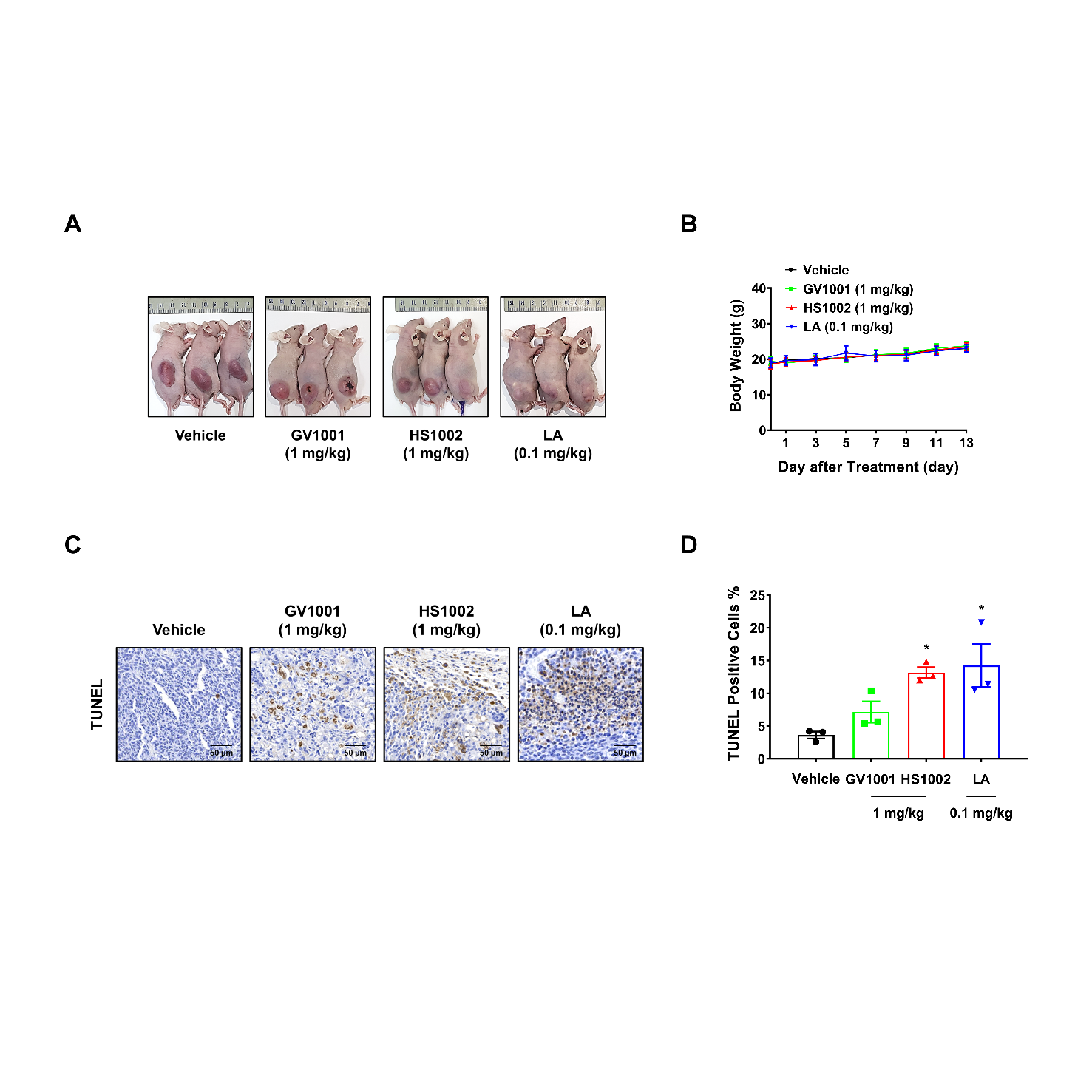


**FIGURE S6 HS1002-induced tumor suppression and apoptosis in LNCaP xenograft models.** (A) Representative images, (B) Body weight change of tumor-implanted mice treated with vehicle, GV1001, HS1002, or LA. (C) Representative images of the TUNEL assay in xenografted LNCaP tumor tissue. Scale bar: 50 µm. (D) TUNEL-positive cells were quantified using ImageJ software. The values represent the means ± SD. **p* < 0.05 vs. the vehicle group.

**
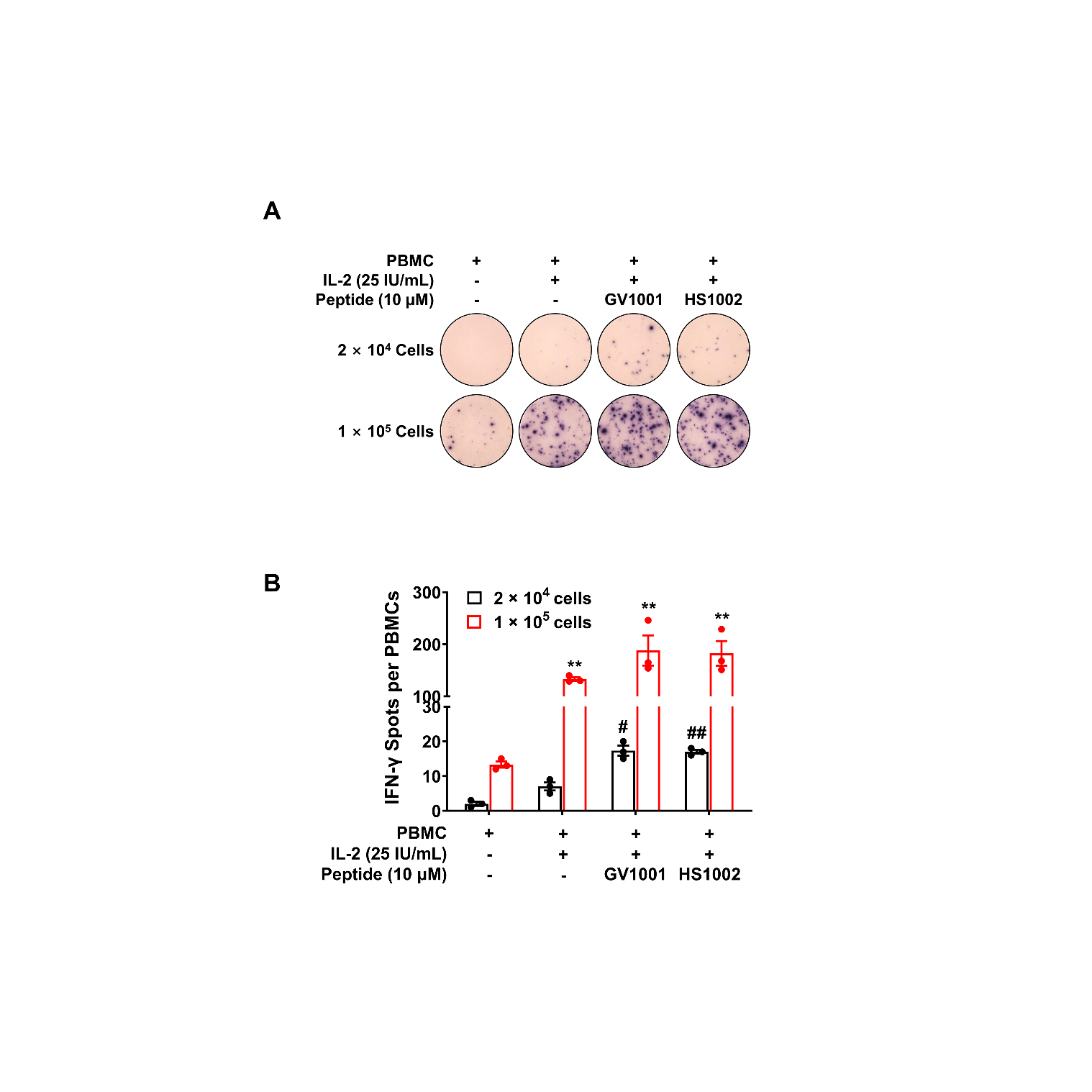
**

**FIGURE S7 Evaluating the immunogenicity of peptides by IFN-γ ELISpot assay.** (A) Representative IFN-γ ELISpot images of PBMC exposed to GV1001 or HS1002 peptides (B) Quantification of the number of spots observed in the ELISpot assay. The values represent the means ± S.D. ***p* < 0.01 vs. the PBMC group; ^#^*p* < 0.05 and ^##^*p* < 0.05 vs. the IL-2 only group.


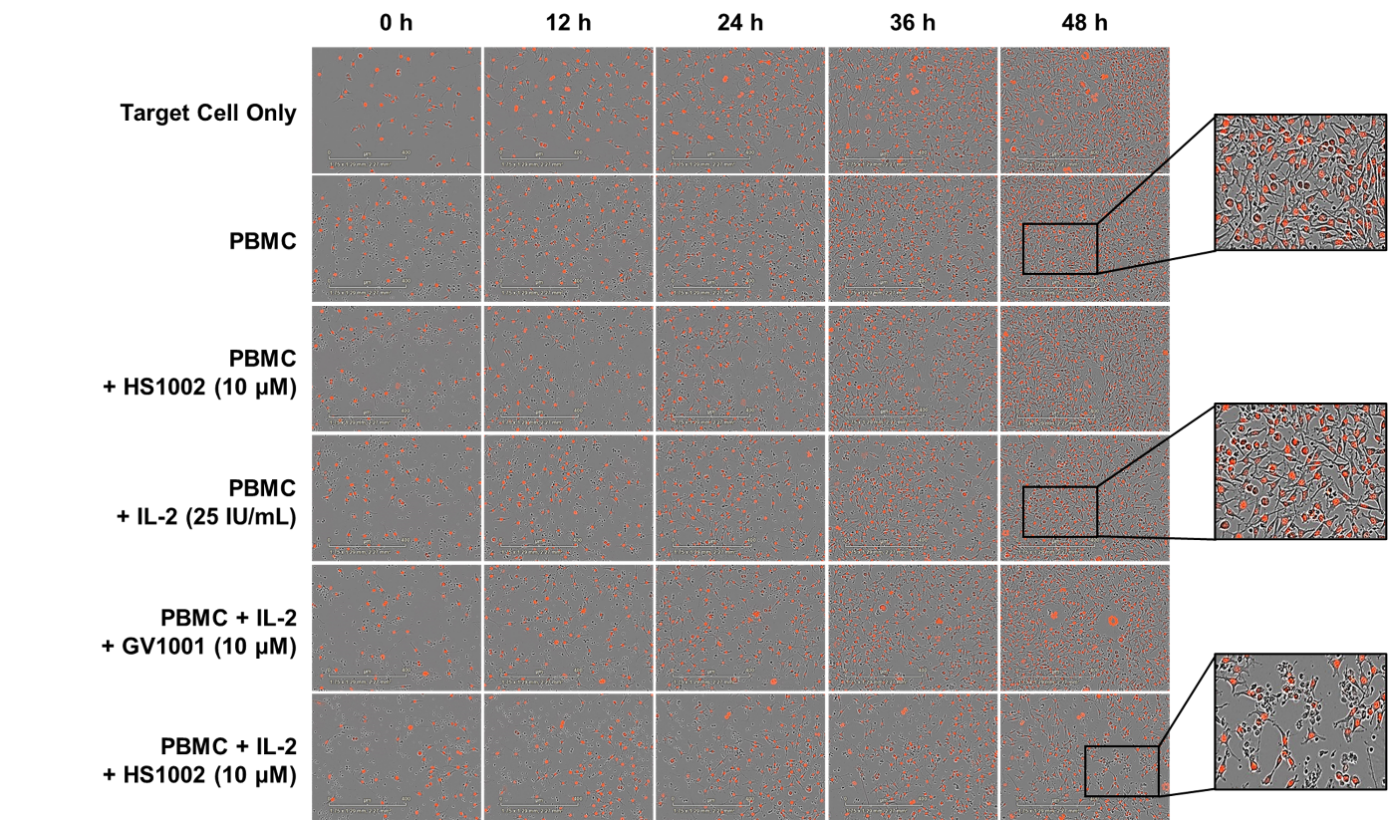


**FIGURE S8 Cytotoxic effects of HS1002/IL-2- or GV1001/IL-2-pretreated PBMCs on LNCaP cells.** LNCaP cells expressing NucLight Red were co-cultured with HS1002/IL-2- or GV1001/IL-2-treated PBMCs for 48 h. Red fluorescence indicates viable LNCaP cells. Scale bar: 400 µm.


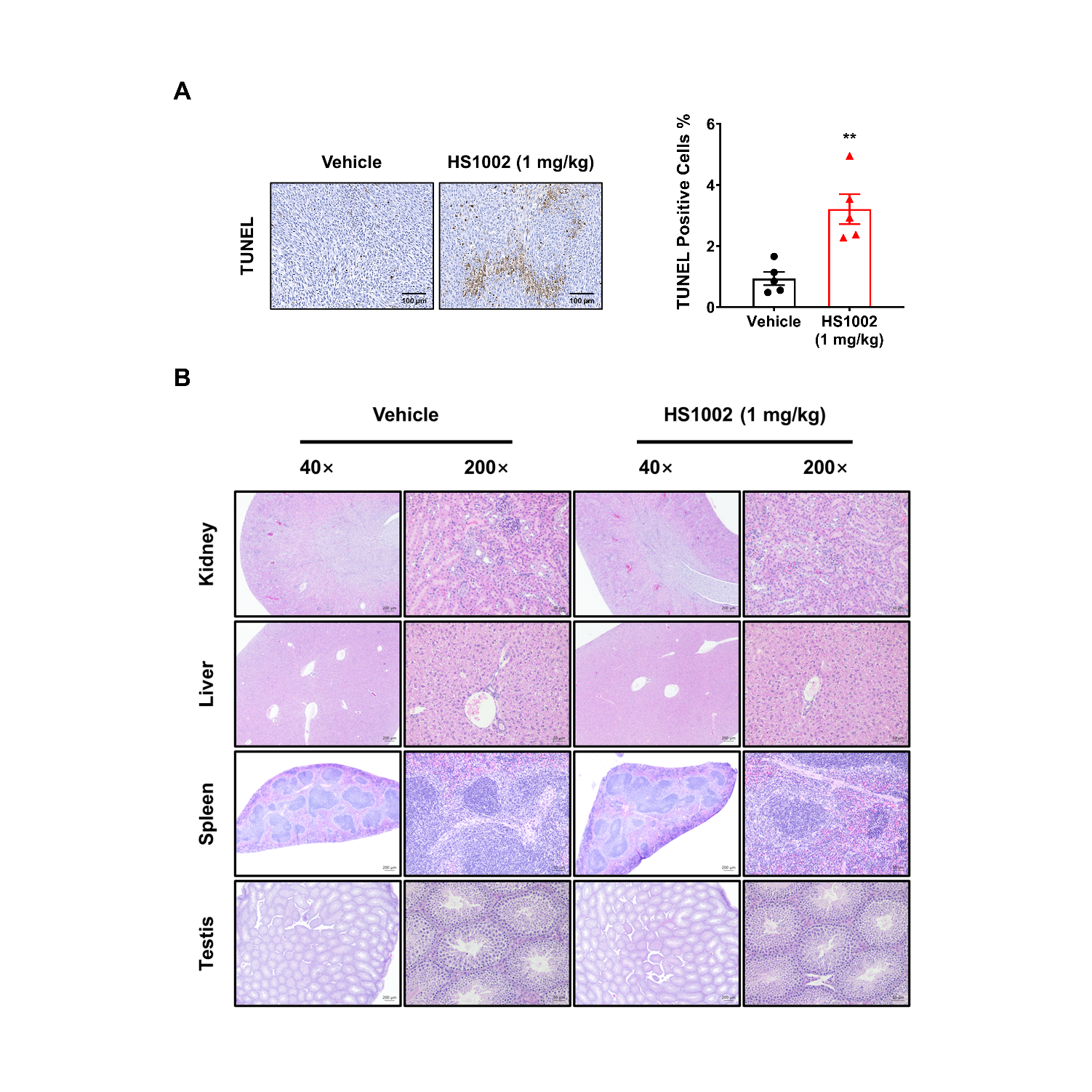


**FIGURE S9 Evaluation of HS1002-induced apoptosis and off-target organ toxicity in MC38 syngeneic mouse models.** (A) TUNEL assay for detection of apoptosis in tumor tissue sections. TUNEL-positive cells were determined by ImageJ software. Scale bar: 100 µm. (B) Representation images of organ sections stained with H&E. The values represent the means ± SD. Scale bar: 200 µm in 40×, 100 µm in 200×. ***p* < 0.01 vs. the vehicle group.


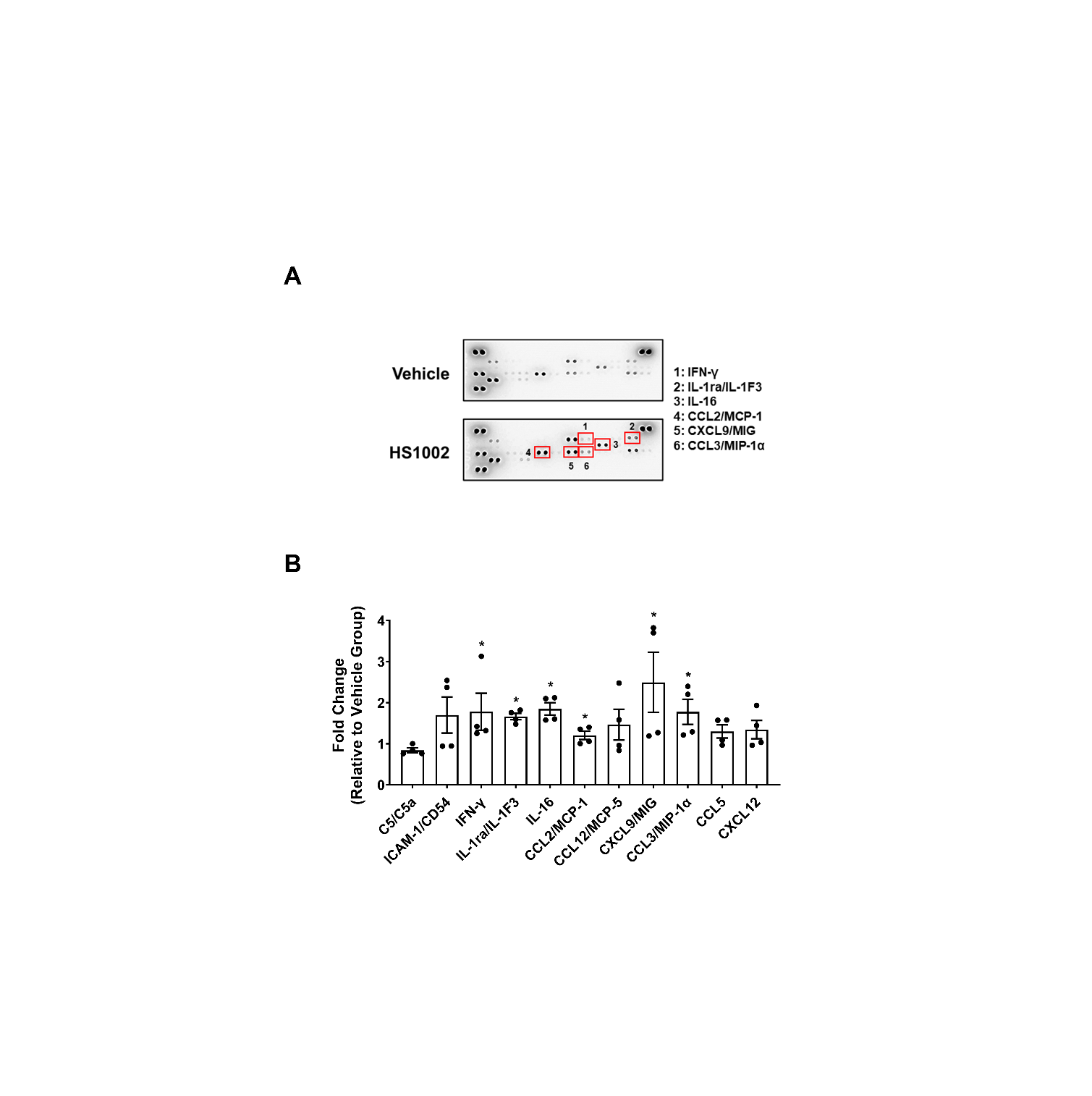


**FIGURE S10 Cytokine profiling in MC38 tumor tissues following HS1002 treatment.** (A, B) Measurement of cytokines in vehicle or HS1002-treated MC38 tumor tissues by cytokine array. The values represent the means ± SD. **p* < 0.05 vs. the vehicle group.

**Supplementary Materials and methods**

Reagents

The peptide was synthesized by Anygen Co. Ltd. (Gwangju, Korea) using a solid-phase synthesis method. Peptide solution was made using Dulbecco's phosphate-buffered saline (DPBS). The culture medium and fetal bovine serum (FBS) were obtained from Gibco (Carlsbad, CA, USA). Primary antibodies against c-Myc, PARP, cleaved PARP, N-cadherin, AKT, p-AKT (Ser473), ERK1/2, p-ERK1/2 (Thr202/Tyr204), mTOR, p-mTOR (Ser2448), Beclin-1, ATG7, and p62 were obtained from Cell Signaling Technology (Danvers, MA, USA). The primary antibodies against hTERT, E-cadherin, Vimentin, Bax, and LC3B were purchased from Abcam (Cambridge, USA). The primary antibodies against β-actin, Bcl-2, and TIMP-1 were purchased from Santa Cruz Biotechnology (Santa Cruz, CA, USA). Horseradish peroxidase (HRP)-conjugated secondary antibodies were purchased from Novus Biologicals (Minneapolis, MN, USA). Z-VAD-FMK and 3-methyladenine (3-MA) were purchased from MedChemExpress (Monmouth Junction, NJ, USA). Fluorochrome-conjugated antibodies were used for flow cytometry: PerCP/Cyanin5.5 anti-CD45 (clone 30-F11), FITC anti-CD8a (clone 53-6.7), APC anti-Granzyme B (clone QA16A02), and PE anti-IFN-γ (XMG1.2) from Biolegend (San Diego, CA, USA).

Cell lines and cell culture

Human prostate cancer cell lines (DU145, LNCaP, PC3, and 22Rv1), breast cancer cell lines (MCF-7 and MDA-MB-231), ovarian cancer cell line (SKOV3), and embryonic kidney cell line (HEK293) were obtained from the American Type Culture Collection (Manassas, VA, USA). All cell lines were grown in Roswell Park Memorial Institute (RPMI) 1640 or Dulbecco's Modified Essential Medium (DMEM), including 10% FBS, 100 μg/mL streptomycin, and 100 U/mL penicillin (WelGENE, Daegu, South Korea) in a humidified environment with 5% CO_2_ at a temperature of 37°C. The pCMV-AC-GFP-GnRHR vector was transfected using Lipofectamine 3000 (Cat. 100022052, Thermo Fisher Scientific, Waltham, MA, USA) to establish GnRHR-overexpressing HEK293 (HEK293-GnRHR) cells. Colonies of geneticin-resistant cells were chosen by adding G418 (800 μg/mL, Sigma) into the growth medium. HEK293-pCMV-AC-GFP vector (HEK293-Vector) cells were used as a mock-transfection group.

siRNA transfection

GnRHR dsiRNA (10 nM; Cat. hs.Ri.GNRHR.13, IDT, Coralville, IA, USA) using Lipofectamine^TM^ RNAiMAX (Cat. 56532, Thermo Fisher Scientific) was employed to knockdown endogenous GnRHR in LNCaP cells. The universal negative control dsiRNA (Cat. 51-01-14-03, IDT) was employed as a negative control.

Prostate tissue microarray

Tissue microarrays (Cat. PR1921c and PR633), consisting of 9 normal human prostate and 110 prostate cancer samples, were obtained from US Biomax Inc. (Rockville, MD, USA). All tissues were collected under HIPAA-compliant protocols, with informed consent from all donors, and fully anonymized. An immunohistochemistry (IHC) analysis was performed to demonstrate hTERT expression in both normal prostate and prostate cancer tissue samples. The tissue microarray underwent incubation with xylene and graded ethanol and was subsequently subjected to boiling in sodium citrate buffer for a duration of 15 min. The slides were exposed to 5% H_2_O_2_ for 15 min, followed by treatment with the hTERT antibody (Cat. ab230527; diluted at a ratio of 1:100; Abcam) at 4°C for 24 h. Slides were incubated with a secondary goat anti-rabbit IgG antibody (VECTOR Laboratories, Burlingame, CA, USA) for 30 min. Subsequently, the slides were incubated with horseradish peroxidase-streptavidin reagent (VECTOR Laboratories) for 30 min. Following treatment with DAB (Dako, Agilent, Santa Clara, CA, USA) and hematoxylin (Dako), the slides were fixed in a mounting solution. The slides were examined under a K1-fluo microscope (Nanoscope Systems, Daejeon, Korea). The hTERT immunostaining intensity was classified using a four-point scale: 0, negative staining; 1, weak intensity; 2, medium intensity; and 3, high intensity. The proportion of hTERT-immunostained cells was quantified, with values ranging from 0 to 100. The overall hTERT immunostaining score, which ranges from 0 to 300, was determined by combining the scores for the percentage of stained cells and staining intensity.

Cytotoxicity assay

Cell viability was determined using the MTT assay (5 mg/mL, Sigma-Aldrich Co., Milan, Italy). The cells were grown in a 96-well plate for 24 h, followed by treatment with each peptide for 72h. At the endpoint, 100 μL of MTT reagent was added and then incubated in the dark at 37°C for 3 h. Absorbance was measured at 540 nm using a VERSA MAX Microplate Reader (Molecular Devices Corp., CA, USA).

Western blot analysis

LNCaP cells were seeded at a density of 2.5 × 10^5^ cells per 100-mm dish, followed by drug treatment for 72 h. Cells and tumor tissues were homogenized using PRO-PREP^TM^ extraction solution (iNtRON Biotechnology, Seongnam, Korea). After incubation, the homogenate was centrifuged at 15,000 × g for 15 min, and the supernatant was collected. An equal amount of protein was isolated using sodium dodecyl sulfate-polyacrylamide gel electrophoresis and transferred onto a polyvinylidene difluoride (PVDF) membrane. The PVDF membranes were subjected to overnight incubation with the primary antibodies at a temperature of 4°C. Subsequently, they were washed with tris-buffered saline and then incubated with HRP-conjugated anti-mouse or anti-rabbit antibodies. The bands were detected using a ChemiDoc imaging system (Bio-Rad, Hercules, CA, USA).

qRT-PCR

The total RNA from the cells was extracted using Qiazol Lysis Reagent (Qiazen, Germantown, MD, USA). A total RNA was converted into cDNA using the Reverse Transcription Master Premix (Elpis Biotech, Daejeon, Republic of Korea). The FastStart Essential DNA Green Master (Roche, Penzberg, Germany) was used to perform quantitative reverse-transcription polymerase chain reaction (qRT-PCR) in a LightCycler 96 Real-Time PCR system (Roche, Penzberg, Germany). The PCR primers used for the *hTERT* gene were 5′-TGACACCTCACCTCACCCAC-3′ (forward) and 5′-CACTGTCTTCCGCAAGTTCAC-3′ (reverse), while for *β-actin*, the primers used were 5′-CCTGGCACCCAGCACAAT-3′ (forward) and 5′-GCCGATCCACACGGAGTACT-3′ (reverse). The 2 ^–ΔΔCt^ method was used to calculate the relative change in gene expression.

Annexin V-FITC binding assay

The Annexin V-FITC binding assay was performed using the Annexin V-FITC staining kit I (BD Biosciences, San Diego, CA, USA). The LNCaP cells were treated with each peptide for 72 h. After the collection of cells, a solution consisting of 5 μL of Annexin V-FITC and propidium iodide (PI) in 100 μL of binding buffer was introduced and then incubated in the dark for 15 minutes. After incubation, the cells were examined using the NovoCyte System (ACEA Biosciences, San Diego, CA, USA).

Acridine orange staining

LNCaP cells were seeded at a density of 2 × 10^4^ cells per confocal dish and allowed to adhere for 24 h before treatment with HS1002 for an additional 72 h. Subsequently, the cells were incubated with acridine orange (1 µg/ml) for 15 minutes, followed by PBS washing to remove the dye. Imaging was performed using a K1-fluo fluorescence microscope (Nanoscope Systems, Daejeon, Korea) at 400× magnification.

Wound healing and Matrigel invasion assay

The effect of peptides on cell migration of LNCaP cells was assessed using the IncuCyte ZOOM™ system (Essen Bioscience, MI, USA). A scratch wound was generated using WoundMaker (Essen Bioscience) after cells reached 100% confluence. Subsequently, the cells were treated with each peptide. Confluency was calculated using the IncuCyte ZOOM^TM^ program. Matrigel invasion assay was conducted using Corning BioCoat Matrigel Invasion Chambers with an 8.0 μm PET Membrane (Cat. 354480; BD Biosciences, Sparks, MD, USA). LNCaP cells were seeded in the top chambers of a serum-free medium with different concentrations of the peptide, whereas the lower chambers were filled with 10% FBS medium. After 24 h of incubation, non-migrating cells in the upper chambers were eliminated using a cotton brush, whereas those in the lower chambers of the membrane were fixed with 4% paraformaldehyde. Following that, the cells were treated with a 0.1% crystal violet solution and manually quantified in random microscopic fields.

Caspase-3/7 activity assay

Caspase-3/7 activity was quantified using the Caspase-Glo® 3/7 Assay System (Cat. G8091, Promega, Madison, WI, USA). The LNCaP cells were plated in 96-well plates at a density of 1.5 × 10^3^ cells/well. Following a 72 h treatment with HS1002, Caspase-Glo® 3/7 reagent (100 μL) was introduced to each well. The plate was subsequently placed in a dark environment and allowed to incubate for 3 h at room temperature. The luminescence was recorded using a microplate luminometer (Thermo Scientific^TM^ Varioskan^TM^ LUX Multimode Microplate Reader).

c-Myc transcription factor assay

The c-Myc Transcription Factor Assay Kit (Cat. ab207200, Abcam) was employed to measure the level of c-Myc transcriptional activation in the nuclear extracts after peptide treatment. LNCaP cells were treated with HS1002 or 10058-F4 for 72 h. Nuclear extracts were obtained using a nuclear extraction kit (Cat. ab113474, Abcam). The protein content was quantified using the Bradford method, which involved mixing 798 μL of Bradford reagent, 200 μL of water, and 2 μL of the protein sample. The absorbance was measured at 595 nm. To assess c-Myc transcriptional activity, 5 μg of nuclear extracts were used, and their absorbance was measured using a spectrophotometer at OD 450 nm.

Intracellular calcium flux assay

Intracellular calcium flux was quantified using a Calcium Flux Assay Kit (Cat. ab233472; Abcam) in response to varying concentrations of each peptide. The percentage increase was determined in relation to the basal fluorescence intensity. All experiments were performed in triplicate.

CRE-luciferase reporter assay

The HEK293-pCMV6-AC-GFP and HEK293-GnRHR cells (4 × 10^4^ cells/well) were grown in 96-well plates and transfected with a luciferase reporter plasmid including a cAMP response element (CRE). Transfected cells were exposed to different concentrations of the peptide for 24 h, and promoter activity was determined using a dual-luciferase reporter assay system (BPS Bioscience). The Renilla and firefly luciferase activities were measured using a microplate luminometer (Thermo Scientific^TM^ Varioskan^TM^ LUX Multimode Microplate Reader). Relative luciferase activity was calculated by normalizing the firefly luciferase activity driven by the Renilla luciferase promoter.

IFN-γ enzyme-linked immunosorbent spot (ELISpot) assay

The enzyme-linked immunosorbent spot (ELISpot) kit (R&D Systems, Minneapolis, USA) was used to measure the production of IFN-γ in peripheral blood mononuclear cells (PBMC). Briefly, different numbers of PBMC were seeded in RPMI-1640 supplemented with 10% FBS and 25 IU/mL interleukin (IL)-2 (Cat. 11011456001, Sigma-Aldrich). Each peptide was added at a concentration of 10 µM and incubated for 24 h. After incubation, the ELISpot plate was incubated with a biotinylated antibody at 4°C overnight. The plate was incubated with streptavidin for 2 h at room temperature. Finally, the spots were developed by adding BCIP (5-bromo-4-chloro-3-indolyl phosphate) and NBT (Nitro blue tetrazolium) solution for 1 h. The colored spots were counted using an ImmunoSpot® analyzer (Cellular Technology Ltd., Columbus, OH).

Histopathological and apoptotic evaluation of tumor tissues

Tumor tissues were fixed in 4% neutral-buffered paraformaldehyde, dehydrated, and embedded in paraffin. Paraffin blocks were sectioned at 4 µm thickness for histological and apoptotic analysis. For general histopathological evaluation, sections were deparaffinized in xylene, rehydrated through graded ethanol, and stained with hematoxylin and eosin (H&E). To assess apoptosis, the DeadEnd™ Colorimetric TUNEL system (Promega Corporation, Madison, WI, USA) was used according to the manufacturer’s instructions. Briefly, tissue sections were dewaxed in 100% xylene, rehydrated in ethanol, and post-fixed with 4% paraformaldehyde. Slides were then treated with proteinase K, hydrogen peroxide (H₂O₂), and TdT reaction mix, and incubated in a humidified chamber at 37°C for 1 h.

Measurement of serum testosterone level

Six-week-old male BALB/c mice were administered with vehicle or 1 mg/kg/day HS1002 for 10 days subcutaneously. Blood samples were collected 5 h after the initial injection and 5 h after the last injection. Serum testosterone concentration was determined using a testosterone ELISA kit (ALPCO Diagnostics, Salem, NH, USA).

Library preparation, sequencing, and data analysis

Total RNA was extracted using QIAzol^TM^ Lysis Reagent (Cat. 79306, Qiagen Sciences, MD, USA). RNA quality was examined using a TapeStation4000 System (Agilent Technologies, Amstelveen, Netherlands), and RNA quantification was conducted using an ND-2000 Spectrophotometer (Thermo Inc., DE, USA). Libraries were constructed from total RNA using the NEBNext Ultra II Directional RNA-Seq Kit (NEW ENGLAND BioLabs, Inc., UK). mRNA was isolated using a Poly(A) RNA Selection Kit (LEXOGEN, Inc., Austria). cDNA synthesis and shearing were performed on the isolated mRNAs. The process of indexing was conducted using Illumina indices 1–12. Enrichment was performed using PCR. Following that, the libraries were assessed using a TapeStation HS D1000 Screen Tape (Agilent Technologies, Amstelveen, Netherlands) to determine the mean size of the fragments. The quantification was performed using a library quantification kit and the StepOne Real-Time PCR System (Life Technologies, Inc., USA). High-throughput sequencing was conducted as paired-end 100 sequencing using NovaSeq 6000 (Illumina, Inc., USA). Quality control of the raw sequencing data was performed using FastQC. FASTX_Trimmer and BBMap were used to exclude adapter and low-quality reads (<Q20). Subsequently, the trimmed reads were aligned to the reference genome using TopHat software. The read count data were analyzed based on the fragments per kilobase per million (FPKM) + geometric normalization method using EdgeR within R. FPKM reads were calculated using Cufflinks. Data mining and graphic visualization were performed using ExDEGA (Ebiogen Inc., Seoul, Korea).
